# Phylogeny, biogeography, and character evolution of *Anaphalis* (Gnaphalieae, Asteraceae)

**DOI:** 10.3389/fpls.2024.1336229

**Published:** 2024-02-07

**Authors:** Xue-Min Xu, He Xu, Zheng Yang, Zhen Wei, Jun-Yu Gu, Dan-Hui Liu, Quan-Ru Liu, Shi-Xin Zhu

**Affiliations:** ^1^ School of Life Sciences, Zhengzhou University, Zhengzhou, China; ^2^ Resource Research Institute, Henan Provincial Third Institute of Resources and Environment Investigation, Zhengzhou, China; ^3^ Xinjiang Institute of Ecology and Geography, Chinese Academy of Sciences, Urumchi, China; ^4^ College of Life Sciences, Beijing Normal University, Beijing, China

**Keywords:** *Anaphalis*, Asteraceae, biogeography, Gnaphalieae, molecular phylogenetic

## Abstract

The HAP clade, mainly including *Helichrysum* Mill, *Anaphalis* DC., and *Pseudognaphalium* Kirp., is a major component of tribe Gnaphalieae (Asteraceae). In this clade, *Anaphalis* represents the largest genus of Asian Gnaphalieae. The intergeneric relationships among *Anaphalis* and its related genera and the infrageneric taxonomy of this genus are complex and remain controversial. However, there are few studies that have focused on these issues. Herein, based on the current most comprehensive sampling of the HAP clade, especially *Anaphalis*, we conducted phylogenetic analyses using chloroplast (cp) genome and nuclear ribosomal DNA (nrDNA) to evaluate the relationships within HAP clade, test the monophyly of *Anaphalis*, and examine the infrageneric taxonomy of this genus. Meanwhile, the morphological characters were verified to determine the circumscription and infrageneric taxonomy system of *Anaphalis*. Additionally, the biogeographical history, diversification processes, and evolution of crucial morphological characters were estimated and inferred. Our phylogenetic analyses suggested that *Anaphalis* is polyphyletic because it nested with *Helichrysum* and *Pseudognaphalium*. Two and four main clades of *Anaphalis* were identified in cp genome and nrDNA trees, respectively. Compared with nrDNA trees, the cp genome trees were more effective for phylogenetic resolution. After comprehensively analyzing morphological and phylogenetic evidence, it was concluded that the achene surface ornamentation and leaf base showed less homoplasy and supported the two *Anaphalis* lineages that were inferred from cp genome. Our biogeographical analyses based on cp genome indicated that HAP clade underwent rapid diversification from late Miocene to Pliocene. The two *Anaphalis* lineages appeared to have originated in Africa, then spread to Western and Southern Asia, and subsequently moved into Southwestern China forming a diversity center. The dispersal patterns of the two *Anaphalis* lineages were different. One dispersed around the world, except in Africa and South America. The other one dispersed to Eastern and Southeastern Asia from the ancestral origin region.

## 1 Introduction

The Gnaphalieae Cass. ex Lecoq & Juill. (Compositae/Asteraceae: Asteroideae) is a large tribe, with ca. 178 genera and ca. 2100 species (Smissen et al., 2020). This tribe has colonized all continents except for Antarctica and concentrates largely in southern Africa and Australia, with smaller diversity centers in the Mediterranean region and South America, while there are fewer taxa in the Northern Hemisphere (Anderberg, 1991; Bayer et al., 2007; Bergh and Linder, 2009; Nie et al., 2016). Based on the latest phylogenetic analyses, two subtribes were included in Gnaphalieae; these were a largely African-endemic Relhaniinae (124 species in 11 genera) and a much enlarged Gnaphaliinae, the latter accounting for more than 90% of the species diversity (ca. 2000 species in 167 genera) and containing six clades, Ifloga, Metalasia, Stoebe, HAP (mainly including *Anaphalis* DC., *Pseudognaphalium* Kirp., and *Helichrysum* Mill), FLAG (mainly including *Filago* Loefl., *Leontopodium* R.Br. ex Cass., *Antennaria* Gaertn., and *Gamochaeta* Wedd.), and Australasian clades (Smissen et al., 2020).

The genus *Anaphalis* represents the largest genus in the Asian Gnaphalieae, with ca. 110 species, and is distributed mainly in tropical and subtropical Asia (Chen et al., 2011; Nie et al., 2013). More than 50% of *Anaphalis* species occur in the Eastern Himalayas and Hengduan Mountains of Southwestern China, and most are endemic to this region (Chen et al., 2011). *Anaphalis* is mainly characterized by subdioecy, hermaphrodite floret sterility, and dimorphic pappus, which have been used as diagnostic characters to circumscribe this genus (Bentham and Hooker, 1873; Mueller, 1889; Beauverd, 1913; Chen et al., 1966; Drury, 1970; Ling, 1979; Anderberg, 1991; Chen et al., 2011). Some *Anaphalis* taxa, such as *A. hancockii* Maxim., *A. lactea* Maxim., and *A. margaritacea* (L.) Benth. & Hook.f., are commonly used in China as aromatic plants or herbal remedies (Ling, 1979; Sun et al., 2001; Yuan et al., 2004; Bao et al., 2009).

The circumscription of *Anaphalis* and its closely related genera is not clear based on morphological characters. Bentham and Hooker (1873) initially defined *Anaphalis* using subdioecy. Subdioecy can be defined as plants differing in the ratio of female to bisexual florets in their capitula, i.e., in some plants their capitula have few hermaphrodite florets while in others hermaphrodite florets predominate. Chen et al. (1966) studied Chinese *Anaphalis* and found that the ratio of female to bisexual florets in capitula was different, suggesting that this genus was evolving from monoecious to heteroecious. Drury (1970) considered subdioecy and capitulum arrangement as important characters distinguishing *Anaphalis* from *Gnaphalium* L. Ling (1979) later utilized subdioecy and pappus characters to differentiate *Anaphalis* from *Antennaria* and *Leontopodium*. Anderberg (1991) also emphasized that subdioecy readily distinguished *Anaphalis* species from closely related genera, such as *Pseudognaphalium*, *Helichrysum*, and *Gnaphalium*. However, Grierson (1972) suggested that the broad concept of subdioecy in *Anaphalis* was questionable, citing evidence from *Anaphalis* species in Sri Lanka with a consistent ratio of hermaphrodite florets in the capitulum, showing no evidence of subdioecy. Another significant character of *Anaphalis* is hermaphrodite floret sterility. Mueller (1889) employed this character to differentiate *Anaphalis* from its closely related genera *Pseudognaphalium* and *Helichrysum*. Beauverd (1913) supported *Anaphalis* as a distinct genus, noting the sterility of hermaphrodite florets in both female-predominant and hermaphrodite-predominant capitula. Ling (1979), however, argued that hermaphrodite floret sterility was not exclusive to *Anaphalis*, citing its presence in *Antennaria* and *Leontopodium* as well. Ling used this character to distinguish these three genera from *Gnaphalium*, *Helichrysum*, and *Phagnalon* Cass. Additionally, Chen et al. (2011) identified the dimorphic pappus as a crucial character distinguishing *Anaphalis* from its related genera *Pseudognaphalium*, *Helichrysum*, and *Xerochrysum* Tzvelev. Consequently, it is evident that there is no definitive diagnostic character for *Anaphalis*.

Recent molecular phylogenetic studies have revealed a close relationship between *Anaphalis*, *Helichrysum*, and *Pseudognaphalium*. However, the monophyly of *Anaphalis* remains uncertain. Molecular phylogenetic analyses by Glenny and Wagstaff (1997) and Breitwieser et al. (1999), focusing on nuclear ribosomal DNA (nrDNA) internal transcribed spacer (ITS), indicated a close association between *Anaphalis* and *Pseudognaphalium*. Additionally, morphological characters, such as style arm cells, anther apices, and corolla and involucral bract hairs, supported the separation of *Anaphalioides* (Benth.) Kirp. from *Anaphalis* (Glenny, 1997; Glenny and Wagstaff, 1997). Later, the phylogenetic analyses by Smissen et al. (2020) suggested *Anaphalioides* should be included in the Australasian clade. A clade containing two *Pseudognaphalium* species and the Southern African species *Helichrysum patulum* D.Don was supported by the analysis of chloroplast (cp) sequences (*trnL* intron, *trnL-trnF*, and *matK*), and this clade was associated with *A. margaritacea* (Ward et al., 2009). Similarly, Galbany-Casals et al. (2010) also suggested a close relationship among the three genera *Helichrysum*, *Anaphalis*, and *Pseudognaphalium*. Therefore, a large portion of the genus *Helichrysum*, along with at least parts of the genera *Anaphalis* and *Pseudognaphalium* (referred to as the HAP clade), has consistently been identified as a clade within the subtribe Gnaphaliinae (Smissen et al., 2020). Based on the nrDNA ITS and external transcribed spacer (ETS) sequences, with a focus on the Eastern Himalayan taxa of *Anaphalis*, Nie et al. (2013) indicated that all *Anaphalis* and *Pseudognaphalium* species nested within *Helichrysum*, forming the well-supported HAP clade, *Anaphalis* appeared close to the Mediterranean-Asian *Helichrysum* clade, but the monophyly of *Anaphalis* was weakly supported. Galbany-Casals et al. (2014) considered the phylogenetic relationships within the HAP clade with the use of two nrDNA markers (ITS and ETS) and two plastid DNA markers (*ndhF* and *rpl32-trnL*) and revealed that *Achyrocline* (Less.) DC., *Anaphalis* and *Pseudognaphalium* nested within the currently broadly defined *Helichrysum*. According to their results, *Anaphalis* was monophyletic based on the nrDNA markers, but in the cpDNA tree, *Anaphalis* was paraphyletic (Galbany-Casals et al., 2014). Consequently, additional studies are required to fully establish the composition of the HAP clade, and additional molecular data and taxa, especially regarding the *Anaphalis* species, should be used to consider the phylogenetic relationships within the HAP clade and examine the monophyly of *Anaphalis*.

The infrageneric taxonomy of *Anaphalis* is complex. Previous morphological and molecular studies have provided substantial insights in the taxonomic relationships within *Anaphalis*. The characters of involucre, capitulum, achene, and leaf have been widely used for the classification of *Anaphalis*. Initially, Hooker (1882) divided *Anaphalis* into two series based on capitulum size. Kitamura (1937) proposed two sections, Sect. *Nagasawae* Kitam. and Sect. *Margaripes* (DC.) Kitam., characterized by monoecy and dioecy, respectively. Borissova (1999) focused on capitulum number and size suggesting Sect. *Anaphalis* Boriss. and Sect. *Polycephales* Boiss. The latter section was further divided into six series (Ser. *Margaripes* (DC.) Boriss., Ser. *Tenuicaules* Boriss., Ser. *Velutinae* Boriss., Ser. *Racemiferae* Boriss., Ser. *Virgatae* Boriss., and Ser. *Scopulosae* Boiss.) (Borissova, 1999). Chen et al. (1966) indicated that *A. bulleyana* (Jeffrey) C.C. Chang was distinct from other *Anaphalis* species because of its obovate involucre, beige and membranous phyllaries, and adhesive hairs. Therefore, they divided *Anaphalis* into two subgenera: Subgen. *Gnaphaliops* Ling (including only *A. bulleyana*) and Subgen. *Anaphalis*. The latter contained two sections: Sect. *Margaripes* (which included seven series: Ser. *Busuae* Ling, Ser. *Margaripes*, Ser. *Oxyphyllae* Ling, Ser. *Sinicae* Ling, Ser. *Suffruticosae* Ling, Ser. *Virgatae*, and Ser. *Xylorhizae* Ling) and Sect. *Anaphalis* (which included three series: Ser. *Flavescentes* Ling, Ser. *Nepalenses* Ling, and Ser. *Pannosae* Ling). Based on the characters of achene surface, 17 taxa of *Anaphalis* from Pakistan were divided into two groups, i.e., sparse or dense papillate-clavate hair (Abid and Qaiser, 2007). Xu et al. (2021) then observed and studied achene micromorphology in 39 *Anaphalis* taxa from China, revealing two types of achene surface ornamentation: reticulate-claviform and ligulate protuberant, which were important for the *Anaphalis* infrageneric taxonomy system. In general, molecular data can provide new insights into taxonomy. However, currently only one study has focused on the molecular phylogeny of *Anaphalis* species. Nie et al. (2013) explored the relationships of 41 *Anaphalis* taxa using two nrDNA markers (ITS and ETS) and indicated that although the monophyly of *Anaphalis* was weakly supported, two clades within the genus were recognized. Furthermore, the phylogenetic relationships within *Anaphalis* corresponded to the shape of leaf base rather than the morphology of capitula and phyllaries, which were usually used for species delimitation and classification in this genus (Nie et al., 2013). Other phylogenetic studies involved only a small number of *Anaphalis* species (Glenny and Wagstaff, 1997; Breitwieser et al., 1999; Ward et al., 2009; Galbany-Casals et al., 2010; Galbany-Casals et al., 2014), which were insufficient for the analysis of the infrageneric taxonomy of this genus. Investigating the infrageneric relationships based on several DNA markers or a relatively small sample size may lead to erroneous and incomplete conclusions. Therefore, additional molecular data and taxa are imperative for a thorough investigation on *Anaphalis* relationships.

The phylogenies are inferred using cp and nuclear genes, which provide a more comprehensive phylogenetic results and can further explore the complex genetic relationships. The chloroplast, with independent genetic material, is responsible for photosynthesis and plays an important role in other aspects of plant physiology and development (Leister, 2003; Xiong et al., 2009; Wicke et al., 2011; Daniell et al., 2016; Tian et al., 2021). Compared with nuclear and mitochondrial genomes, cp genomes evolve at relatively moderate rates and has significantly contributed to phylogenetic and taxonomic studies (Dong et al., 2013; Twyford and Ness, 2017; Jiang et al., 2020; Pascual-Díaz et al., 2021; Wang et al., 2021; Zhang et al., 2021b; Chu et al., 2022; Han et al., 2022; Xu et al., 2023a). However, there are currently only 30 complete cp genomes from the HAP clade (*Anaphalis*, 2; *Helichrysum*, 4; *Pseudognaphalium*, 24) that have been published in the National Center for Biotechnology Information (NCBI) database (https://www.ncbi.nlm.nih.gov/). Therefore, additional cp genome data must be generated and analyzed to reveal the intergeneric relationships of the HAP clade and the infrageneric relationships of *Anaphalis*. Compared with genomic data, DNA barcodes, such as *trnL-F*, *rbcL*, *matK*, *trnK-matK*, *psbA-trnH*, ITS, and ETS, are more readily available and can still be used to solve many taxonomic problems. Among these, ITS and ETS are important nrDNA markers that are widely used in phylogenetic studies (Kuzmanović et al., 2017; Vicent et al., 2017; Zhou and Zhang, 2017; Carrizo García et al., 2018; Asanuma et al., 2019; Pirani et al., 2020; Tan et al., 2020; Hashim et al., 2021; Liao et al., 2023). ITS and ETS are effective in discriminating closely related species because of their rapid evolutionary rates and higher interspecies variation (Li et al., 2011). Additionally, the incongruent phylogenetic relationships between the cp and nuclear genes suggest the existence of hybridization, incomplete lineage sorting, and/or interspecific introgression (Yang et al., 2013; Gatesy et al., 2019). Therefore, complex genetic relationships can be revealed through phylogenetic analyses based on the cp and nuclear genes. However, few studies have considered the intergeneric relationships of the HAP clade and the infrageneric relationships of *Anaphalis* based on both cp and nuclear genes.

In this study, we used the most comprehensive samples of the HAP clade, with a particular emphasis on *Anaphalis* taxa. We constructed phylogenetic trees using cp genomes and nrDNA. The objectives of this study were to (1) reconstruct a phylogeny of the HAP clade to analyze the intergeneric relationships of this clade and test the monophyly of *Anaphalis*; (2) resolve the relationships within *Anaphalis* and test previous morphological classifications of this genus; (3) estimate the biogeographical history and diversification processes of *Anaphalis*; and (4) infer the evolution of the crucial morphological characters.

## 2 Materials and methods

### 2.1 Taxon sampling

The sequences used in this study include both new and previously published sequences. Leaf material from 77 individuals representing 63 taxa (52 *Anaphalis*, 2 *Euchiton* Cass., 1 *Gnaphalium*, 2 *Helichrysum*, 3 *Pseudognaphalium*, 1 *Xerochrysum*, and 2 *Scorzonera* L.) was obtained. Most of the plant material used in this study was collected by our research group from natural populations in China. The voucher specimens were deposited in the herbarium of Zhengzhou University (ZZU; Zhengzhou, China). The plant material from a few taxa was obtained from the herbarium specimens of E (Royal Botanic Garden Edinburgh Herbarium, Edinburgh, UK), PE (Institute of Botany, Chinese Academy of Sciences, Beijing, China), and XJBI (Xinjiang Institute of Ecology and Geography, Chinese Academy of Sciences, Urumchi, China). Detailed information regarding the voucher specimens is provided in **Table 1**. Furthermore, the cp genome, ITS and ETS sequences (51 cp genomes, 744 ITS, and 836 ETS) of Gnaphalieae and related taxa were acquired from the NCBI database and are listed in **Supplementary Tables S1**, **S2**, respectively. Based on the phylogenetic results of Fu et al. (2016); Huang et al. (2016), Panero and Crozier (2016), Mandel et al. (2019), and Zhang et al. (2021a), *Scorzonera humilis*, *S. radiata*, *Cichorium intybus* L., *Lactuca praevia* C.D.Adams, and *L. sativa* L. were used as outgroups.

**Table 1:** Voucher specimens and location information, and accession numbers of cp genomes, ITS, and ETS sequences from NCBI database.

Taxa	Accession numbers			Voucher specimen	Location
	Cp	ITS	ETS		
*Anaphalis acutifolia* Hand.-Mazz.	OR727193	OR700107	OR711267	D. E. Boufford et al. 31928 (ZZU)	Riwoqe, Xizang, China
*A. adnata* Wall. ex DC.	OR727259	OR700173	OR711333	B. Z. Xiao 4748 (PE)	Yizhang, Hunan, China
*A. aureopunctata* Lingelsh. & Borza	OR727194	OR700108	OR711268	D. E. Boufford et al. 38255 (ZZU)	Xiaojin, Sichuan, China
*A. aureopunctata* Lingelsh. & Borza	OR727195	OR700109	OR711269	D. E. Boufford et al. 38963 (ZZU)	Rangtang, Sichuan, China
*A. aureopunctata* Lingelsh. & Borza	OR727196	OR700110	OR711270	D. E. Boufford et al. 39694 (ZZU)	Maerkang, Sichuan, China
*A. aureopunctata* var. *plantaginifolia* Y.L. Chen	OR727197	OR700111	OR711271	S. X. Zhu et al. DS13531 (ZZU)	Baoxing, Sichuan, China
*A. aurora* Rech.f. & Edelb.	OR727198	OR700112	OR711272	S. B. Lyon 8065 (E)	Yasin Darkot, Pakistan
*A. bicolor* (Franch.) Diels	OR727199	OR700113	OR711273	D. E. Boufford et al. 36420 (ZZU)	Dege, Sichuan, China
*A. brevifolia* DC.	OR727200	OR700114	OR711274	D. Clayton 5515 (E)	Badulla, Uva, Sri Lanka
*A. brevifolia* DC.	OR727201	OR700115	OR711275	F. W. Gould et al. 13833 (E)	Badulla, Uva, Sri Lanka
*A. bulleyana* (Jeffrey) C.C. Chang	OR727202	OR700116	OR711276	S. X. Zhu et al. DS11451 (ZZU)	Lijiang, Yunnan, China
*A. busua* DC.	OR727203	OR700117	OR711277	S. X. Zhu et al. DS11458 (ZZU)	Lijiang, Yunnan, China
*A. busua* DC.	OR727204	OR700118	OR711278	S. X. Zhu et al. DS13351 (ZZU)	Mianning, Sichuan, China
*A. cavei* Chatterjee	OR727205	OR700119	OR711279	T. Wraber 228 (E)	Kumbhakarna Himal, Nepal
*A. cavei* Chatterjee	OR727206	OR700120	OR711280	Briggs 46759 (E)	Taplejung, Nepal
*A. chlamydophylla* Diels	OR727207	OR700121	OR711281	D. E. Boufford et al. 37391 (ZZU)	Daocheng, Sichuan, China
*A. chungtienensis* F.H. Chen	—	OR700122	OR711282	S. X. Zhu et al. DS13594 (ZZU)	Shangrila, Yunnan, China
*A. cinerascens* Y. Ling & W. Wang	OR727208	OR700123	OR711283	S. X. Zhu et al., 1907351 (ZZU)	Deqin, Yunnan, China
*A. contorta* Hook. f.	OR727209	OR700124	OR711284	S. X. Zhu et al. DS11511 (ZZU)	Xinping, Yunnan, China
*A. contorta* Hook. f.	OR727210	OR700125	OR711285	S. X. Zhu et al. DS13552 (ZZU)	Mianning, Sichuan, China
*A. contortiformis* Hand.-Mazz.	OR727211	OR700126	OR711286	S. X. Zhu et al. DS11523 (ZZU)	Xinping, Yunnan, China
*A. delavayi* (Franch.) Diels	OR727212	OR700127	OR711287	D. E. Boufford et al. 37286 (ZZU)	Xinlong, Sichuan, China
*A. elegans* Y. Ling	OR727213	OR700128	OR711288	S. X. Zhu et al. DS11438 (ZZU)	Shangrila, Yunnan, China
*A. elegans* Y. Ling	OR727214	OR700129	OR711289	S. X. Zhu et al. DS13580 (ZZU)	Muli, Sichuan, China
*A. flaccida* Y. Ling	OR727215	OR700130	OR711290	D. E. Boufford et al. 35858 (ZZU)	Yajiang, Sichuan, China
*A. flavescens* Hand.-Mazz.	OR727216	OR700131	OR711291	D. E. Boufford et al. 39275 (ZZU)	Rangtang, Sichuan, China
*A. gracilis* Hand.-Mazz.	OR727217	OR700132	OR711292	D. E. Boufford et al. 29966 (ZZU)	Baxoi, Xizang, China
*A. hancockii* Maxim.	OR727218	OR700133	OR711293	D. E. Boufford et al. 39171 (ZZU)	Rangtang, Sichuan, China
*A. hancockii* Maxim.	OR727219	OR700134	OR711294	X. M. Xu et al. QH001 (ZZU)	Datong, Qinghai, China
*A. hellwigii* Warb.	OR727220	OR700135	OR711295	W. Takeuchi 5889 (E)	Eastern Highlands, Papua New Guinea
*A. hymenolepis* Y. Ling	OR727221	OR700136	OR711296	D. E. Boufford et al. 41543 (ZZU)	Gongjue, Xizang, China
*A. lactea* Maxim.	OR727222	OR700137	OR711297	D. E. Boufford et al. 39074 (ZZU)	Rangtang, Sichuan, China
*A. larium* Hand.-Mazz.	OR727223	OR700138	OR711298	S. X. Zhu et al. DS11434 (ZZU)	Deqin, Yunnan, China
*A. likiangensis* (Franch.) Y. Ling	OR727224	OR700139	OR711299	S. X. Zhu et al., 1907201 (ZZU)	Shangrila, Yunnan, China
*A. margaritacea* (L.) Benth. & Hook. f.	OR727225	OR700140	OR711300	D. E. Boufford et al. 40183 (ZZU)	Ruoergai, Sichuan, China
*A. margaritacea* (L.) Benth. & Hook. f.	OR727226	OR700141	OR711301	X. M. Xu et al. 150802037 (ZZU)	Jiyuan, Henan, China
*A. margaritacea* var. *angustior* (Miq.) Nakai	OR727227	OR700142	OR711302	M. Togashi 46740 (E)	Yamanashi, Japan
*A. muliensis* (Hand.-Mazz.) Hand.-Mazz.	OR727228	OR700143	OR711303	S. X. Zhu et al. DS11436 (ZZU)	Shangrila, Yunnan, China
*A. nepalensis* (Spreng.) Hand.-Mazz.	OR727229	OR700144	OR711304	D. E. Boufford et al. 41939 (ZZU)	Jiangda, Xizang, China
*A. nubigena* DC.	OR727230	OR700145	OR711305	A. R. Brown et al. 62 (E)	Ladakh, India
*A. pachylaena* F.H. Chen & Y. Ling	OR727231	OR700146	OR711306	S. X. Zhu et al. DS13590 (ZZU)	Litang, Yunnan, China
*A. pannosa* Hand.-Mazz.	OR727232	OR700147	OR711307	S. X. Zhu et al. DS11433 (ZZU)	Deqin, Yunnan, China
*A. racemifera* Franch.	OR727233	OR700148	OR711308	L. Osbourne 470 (E)	Jalal-Abad Oblast, Kyrgyztan
*A. rhododactyla* W.W. Sm.	OR727234	OR700149	OR711309	S. X. Zhu et al. DS13591 (ZZU)	Litang, Sichuan, China
*A. roseoalba* Krasch.	OR727235	OR700150	OR711310	S. Dixon 46757 (E)	Chitral District, Pakistan
*A. royleana* DC.	OR727236	OR700151	OR711311	I. W. J. Sinclair et al. 5617 (E)	Punakha District, Bhutan
*A. sinica* Hance	OR727237	OR700152	OR711312	D. E. Boufford et al. 29691 (ZZU)	Baxoi, Xizang, China
*A. souliei* Diels	OR727238	OR700153	OR711313	S. X. Zhu et al. DS13576 (ZZU)	Muli, Sichuan, China
*A. stenocephala* Y. Ling & Shih	OR727239	OR700154	OR711314	D. E. Boufford et al. 37044 (ZZU)	Baiyu, Sichuan, China
*A. subdecurrens* Gamble	OR727240	OR700155	OR711315	N. Balakrishnan 46756 (E)	Nuwara Eliya, Central Province, Sri Lanka
*A. suffruticosa* Hand.-Mazz.	OR727241	OR700156	OR711316	D. E. Boufford et al. 33158 (ZZU)	Jiulong, Sichuan, China
*A. surculosa* (Hand.-Mazz.) Hand.-Mazz.	OR727242	OR700157	OR711317	D. E. Boufford et al. 36266 (ZZU)	Xinlong, Sichuan, China
*A. szechuanensis* Y. Ling & Y.L. Chen	OR727243	OR700158	OR711318	D. E. Boufford et al. 37371 (ZZU)	Daocheng, Sichuan, China
*A. tenella* DC.	OR727244	OR700159	OR711319	Curzon 67A (E)	Taplejung, Nepal
*A. tibetica* Kitam.	OR727245	OR700160	OR711320	D. E. Boufford et al. 32092 (ZZU)	Riwoqe, Xizang, China
*A. triplinervis* Sims ex C.B. Clarke	OR727246	OR700161	OR711321	D. E. Boufford et al. 35925 (ZZU)	Yajiang, Sichuan, China
*A. virens* C.C. Chang	OR727247	OR700162	OR711322	D. E. Boufford et al. 32134 (ZZU)	Riwoqe, Xizang, China
*A. virgata* Thomson ex C.B. Clarke	OR727248	OR700163	OR711323	C. G. Wilson et al. 1375 (E)	Badakhshan-Wakhan, Afghanistan
*A. virgata* Thomson ex C.B. Clarke	OR727249	OR700164	OR711324	E. Reiser 46761 (E)	Biafo, Pakistan
*A. viridis* H.A. Cummins	OR727250	OR700165	OR711325	S. X. Zhu et al. DS11428 (ZZU)	Deqin, Yunnan, China
*A. xylorhiza* Sch. Bip. ex Hook. f.	OR727251	OR700166	OR711326	D. E. Boufford et al. 41631 (ZZU)	Gongjue, Xizang, China
*A. yunnanensis* (Franch.) Diels	OR727252	OR700167	OR711327	D. E. Boufford et al. 41728 (ZZU)	Jiangda, Xizang, China
*Euchiton involucratus* (G. Forst.) Holub	OR727253	OR700168	OR711328	J. H. Liu et al. 294 (PE)	Miaoli, Taiwan, China
*E. japonicus* (Thunb.) Holub	OR727254	OR700169	OR711329	Y. L. Chen PE0055515 (PE)	Jiangkou, Guizhou, China
*Gnaphalium polycaulon* Pers.	OR727255	—	—	S. X. Zhu et al. ZSX1910094 (ZZU)	Chengdu, Sichuan, China
*Helichrysum argyrophyllum* DC.	OR727256	OR700170	OR711330	Z. Li et al. L001 (ZZU)	Zhengzhou, Henan, China
*H. argyrophyllum* DC.	OR727257	OR700171	OR711331	Z. Li et al. L002 (ZZU)	Zhengzhou, Henan, China
*H. italicum* (Roth) G. Don	OR727258	OR700172	OR711332	Z. Li et al. L003 (ZZU)	Zhengzhou, Henan, China
*Pseudognaphalium affine* (D. Don) Anderb.	OR727260	OR700174	OR711334	D.E.Boufford et al. 37610 (ZZU)	Wenxian, Gansu, China
*P. affine* (D. Don) Anderb.	OR727261	OR700175	OR711335	S. X. Zhu et al. ZSX1907007 (ZZU)	Kunming, Yunnan, China
*P. hypoleucum* (DC.) Hilliard & B.L. Burtt	OR727262	OR700176	OR711336	Jinfoshan Exped. 2234 (PE)	Nanchuan, Sichuan, China
*P. hypoleucum* (DC.) Hilliard & B.L. Burtt	OR727263	OR700177	OR711337	X. M. Xu et al. BNU2019SC273 (ZZU)	Leibo, Sichuan, China
*P. hypoleucum* (DC.) Hilliard & B.L. Burtt	OR727264	OR700178	OR711338	X. M. Xu et al. BNU2019XZ300 (ZZU)	Chayu, Xizang, China
*P. luteoalbum* (L.) Hilliard & B.L. Burtt	OR727265	OR700179	OR711339	E. Bisset 299 (E)	Yeman
*Scorzonera humilis* L.	OR727266	OR700180	OR711340	Anonymous 2244 (XJBI)	Austria
*S. radiata* Fisch. ex Fisch.	OR727267	OR700181	OR711341	Anonymous 10484 (XJBI)	Habahe, Xinjiang, China
*Xerochrysum bracteatum* (Vent.) Tzvelev	OR727268	OR700182	OR711342	F. Zhao 445 (PE)	Beijing, China

“—” means “no data”.

### 2.2 DNA extraction and sequencing, cp genome and nrDNA assembly

We used a modified cetyltrimethyl ammonium bromide (CTAB) method to extract high-quality DNA (Doyle and Doyle, 1987), which was then purified using the Wizard^®^ DNA cleanup system (Promega, Madison, WI, USA). DNA quality was assessed using a NanoDrop spectrophotometer (Thermo Scientific, Carlsbad, CA, USA), and integrity was evaluated through electrophoresis on a 1% (w/v) agarose gel. A DNA library was prepared using the NEB Next Ultra DNA Library Prep Kit for Illumina (NEB, USA). Libraries for paired-end 150-bp sequencing were analyzed on an Illumina NovaSeq 6000 platform (Novogene Co., Ltd., Tianjin, China), to generate approximately 10 GB of data for each sample. Raw reads were filtered using SOAPnuke to remove sequencing adaptors and low-quality bases (Chen et al., 2018). The filtered reads were assembled using GetOrganelle (Jin et al., 2020) with a range of 21, 45, 65, 85, and 105 k-mers for plastomes, and 35, 85, and 115 k-mers for nrDNA. Subsequently, the ITS and ETS sequences were uploaded onto the NCBI database (the accession numbers are listed in **Table 1**).

### 2.3 Cp genome annotation

The plastome sequences were first annotated using Geneious Prime 2020.1.2 (https://www.geneious.com) by referring to the cp genome sequences of *A. sinica* Hance (KX148081), *A. margaritacea* var. *yedoensis* Ohwi (LC656264), and *P. affine* (D.Don) Anderb. (MN541094). The annotations of protein-coding sequences were then manually checked based on the open reading frame. Transfer RNA genes were verified using the online tRNAscan-SE tool with default settings (Lowe and Chan, 2016). All annotated cp genome sequences were deposited in the NCBI database (accession numbers are listed in **Table 1**).

### 2.4 Phylogenetic analysis

Phylogenetic trees were constructed based on four matrices: complete cp sequences, ITS, ETS, and the concatenated sequences of ITS and ETS. A total of 127 cp genomes (including 63 *Anaphalis* samples representing 52 taxa), 820 ITS sequences (including 248 *Anaphalis* samples representing 67 taxa), and 912 ETS sequences (including 213 *Anaphalis* samples representing 67 taxa) were involved in our phylogenetic analyses. Only the nrDNA sequences from the same individual were concatenated, and detailed information is shown in **Supplementary Table S2**. The online version of MAFFT was used to align the datasets (Katoh et al., 2019). The matrix characteristics were analyzed using MegaX (Kumar et al., 2018). The alignments of cp genome, ITS, and ETS sequences are provided in the **Supplementary Material**. The phylogenetic analyses were performed using the maximum likelihood (ML) and Bayesian inference (BI) methods via IQ-TREE v1.6.12 and MrBayes 3.2.2, respectively (Ronquist et al., 2012; Nguyen et al., 2015). The best-fitting model of nucleotide substitutions was determined using ModelFinder in PhyloSuite v1.2.2 (Zhang et al., 2020). The ML analyses were performed using IQ-TREE with 1,000 bootstrap replicates. The BI analysis was run for 50,000,000 generations and sampled every 5,000 generations, and the first 25% of the trees were discarded as burn-in. Trees were selected based on a 50% majority-rule consensus to estimate the posterior probabilities. The effective sample size (ESS, >200) was determined using Tracer v1.7 (Rambaut et al., 2018). Reconstructed trees were visualized using Figtree v1.4.2 (Rambaut, 2014) and TreeGraph 2 (Stöver and Müller, 2010).

### 2.5 Divergence time estimates

Divergence time analyses based on the cp genome matrix were performed using the BEAST v2.6.7 program (Bouckaert et al., 2019), with the Gamma site + GTR nucleotide substitution model, the yule model for tree priors, and relaxed clock log-normal model. Two nodes were calibrated: (1) The crown age of tribe Cichorieae Lam. & DC. (including *Cichorium* L., *Scorzonera*, and *Lactuca* L.) was calibrated based on the oldest reported *Cichorium intybus* type pollen fossil (early Miocene, 22.0–28.4 Mya), which was widely distributed in all Cichorieae subtribes except in Scorzonerinae (Hochuli, 1978; Tremetsberger et al., 2013; Kilian et al., 2017); (2) The earliest *Ambrosia*-type pollen fossil (22–30 Mya) from the Beaverhead Basin flora of Montana was used to calibrate the crown age of tribe Tageteae (including *Ambrosia* L., *Helianthus* L., and *Tagetes* L.) (Becker, 1969; Leopold and MacGinitie, 1972; Graham, 1996; Bergh and Linder, 2009). A Markov chain Monte Carlo (MCMC) analysis was run for 100 million generations, with sampling every 10,000 generations. Other parameters were left at the default values. The BEAST output files were examined through Tracer v1.7 (Rambaut et al., 2018) to evaluate the convergence and ensure that ESS values for all parameters were adequate and > 200. We combined the treefiles (burn-in 20%) in Logcombiner v2.6.7, and used Treeannotator v2.6.7 to produce the maximum clade credibility (MCC) tree showing mean divergence time estimates with 95% highest posterior density (HPD) intervals. The final result was visualized using Figtree v1.4.2 (Rambaut, 2014).

### 2.6 Diversification through time

A Bayesian analysis of macroevolutionary mixtures (BAMM v2.5.0) was performed to analyze the speciation and extinction dynamics of HAP clade (Rabosky et al., 2014a). The appropriate priors on the consensus tree were estimated using the “setBAMMpriors” function in the BAMMtools package in R Studio (Rabosky et al., 2014b). The parameters of “PRIORS” were as follows: expectedNumberOfShifts = 1; lambdaInitPrior = 0.848; lambdaShiftPrior = 0.069; muInitPrior = 0.848; and lambdaIsTimeVariablePrior = 1. The analysis was run for 10 million generations, and the MCMC chain was sampled every 1000 generations. Finally, using BAMMtools, we traced the specific clade diversification and plotted the diversification rate (speciation, extinction, and net diversification rates) through time, after discarding 10% of the generations (MCMC) as burn-in. Further, we constructed the semi-logarithmic lineage through time (LTT) plots of HAP clade using the “phytools” package in R studio (Revell, 2012) to visualize temporal variations in diversification rates (Mahler et al., 2010). Three thousand trees were randomly sampled from the converged BEAST trees of the HAP clade and were used to calculate the 95% credibility interval.

### 2.7 Ancestral area reconstruction

The natural distribution data of taxa in this study was assessed based on the Global Biodiversity Information Facility database (https://www.gbif.org/) and various floras (e.g., Hooker, 1882; Leopold and MacGinitie, 1972; Ling, 1979; Borissova, 1999; Chen et al., 2011). The biogeographical area codes of HAP clade were modified according to those of Gnaphalieae from Nie et al. (2016). Asia was re-coded, considering the highest species richness and endemism of *Anaphalis*. Seven biogeographical areas were coded as follows: (A) North America; (B) Europe to Western Asia; (C) Africa; (D) SW China; (E) South Asia; (F) Eastern and Southeastern Asia; (G) New Guinea, Australia, New Zealand, and Pacific islands. The ancestral area reconstruction was performed using the tree files generated by BEAST under the Statistical Dispersal-Vicariance-Analysis (S-DIVA) model in RASP v4.2 using the default settings (Yu et al., 2020). Random 1000 BEAST output trees were used and the maximum number of individual unit areas was set to four. To reduce geographical irrelevance from distant phylogenetic relationships, we only retained samples of the HAP clade in posterior trees and MCC tree. The analysis was applied allowing up to four areas per node.

### 2.8 Characteristic analyses of morphology

According to the results of Nie et al. (2013) and Xu et al. (2021), we selected two morphological characters to study their evolution in *Anaphalis*. These two characters and their states were achene surface ornamentation (reticulate-claviform, and ligulate protuberant) and leaf base (decurrent, non-decurrent, and cordate). The characteristics information of achene surface ornamentation and leaf base are provided in **Supplementary Table S3**. Ancestral state reconstruction and stochastic character mapping were performed in R Studio v4.1, using the make.simmap commands under the ER model (equal-rates model) in “phytools” package (Huelsenbeck et al., 2003; Paradis et al., 2004; Bollback, 2006; Revell, 2012), The tree file (MCC tree) generated by BEAST was used to analyze and only focused on *Anaphalis*. Stochastic mapping was simulated 200 times, and posterior density tree was plotted in “Phytools” package (Revell, 2012; O’Reilly and Donoghue, 2018).

## 3 Results

### 3.1 Phylogenetic relationships

A total of 228 sequences were newly generated in this study (including 76 cp genomes, 76 ITS, and 76 ETS). The sequence characteristics and nucleotide substitution models for ML and BI phylogenetic analyses of different datasets (complete cp genomes, ITS, ETS, and concatenated ITS and ETS sequences) are presented in **Supplementary Table S4.**

**Figure 1:**
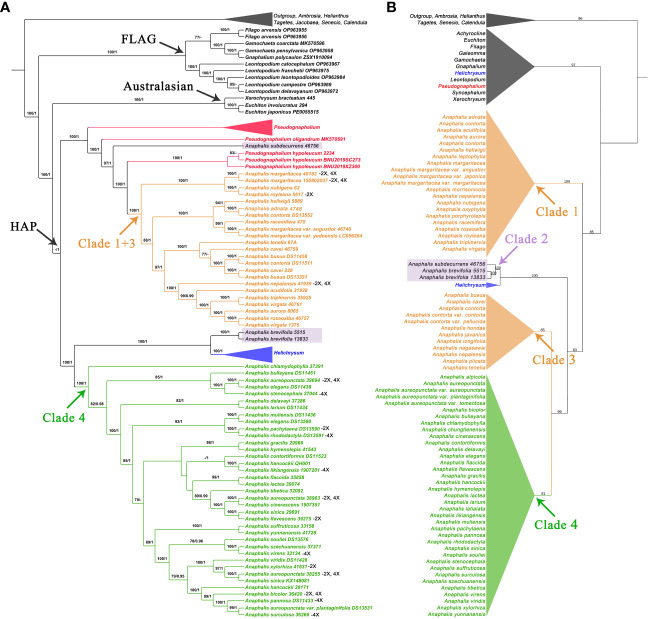
Comparison of phylogenetic trees inferred from the complete chloroplast genomes and the concatenated sequences of ITS and ETS. The skeletal phylogenetic trees of the HAP clade and its closely related genera are showed emphasizing the mainly clades: **(A)** ML tree, inferred from the complete chloroplast genomes, with bootstrap values of ML and posterior probabilities of BI shown at each node. Bootstrap values higher than 70 and posterior probabilities higher than 0.90 are indicated on branches. “-” means that the bootstrap; **(B)** ML tree, inferred from the concatenated sequences of ITS and ETS, with bootstrap values of ML shown at each node.

In the phylogenetic trees inferred using complete cp genomes (**Figure 1A** and **Supplementary Figure 1**), the topologies of BI and MP trees were consistent (**Supplementary Figure 1**). In HAP clade, *Anaphalis* was polyphyletic and nested with *Pseudognaphalium* and *Helichrysum.* Four clades, Clade 1 + 3 [18 taxa, ML bootstrap value (BS) = 100, Bayesian posterior probability (PP) = 1], Clade 4 (32 taxa, BS = 100, PP = 1), and two small clades (each with only one species, *A. subdecurrens* Gamble and *A. brevifolia* DC., respectively), were recognized in *Anaphalis*. Clade 1 + 3 and *A. subdecurrens* were nested with *Pseudognaphalium*, and Clade 4 and *A. brevifolia* were nested with *Helichrysum*.

**Figure 2:**
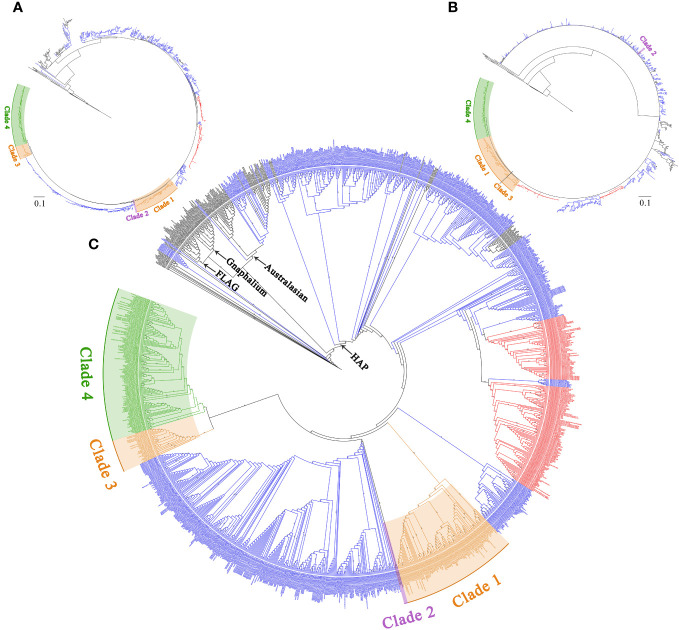
The complete phylogenetic trees of the HAP clade and its closely related genera are inferred from the concatenated sequences of ITS and ETS. **(A)** Topology of the ML tree. **(B)** Topology of the BI tree. **(C)** ML tree, with bootstrap values of ML shown at each node. Bootstrap values higher than 70 are indicated on branches.

In the nrDNA trees (**Figures 1B**, **2** and **Supplementary Figures S2**-**S6**), the topologies of BI and MP trees were inconsistent. Additionally, the phylogenetic trees inferred from different nrDNA matrices (ITS, ETS, and concatenated ITS and ETS sequences) were also inconsistent. The genera sampled in this study, such as *Pseudognaphalium*, *Anaphalis*, *Achyrocline*, *Gnaphalium*, and *Xerochrysum*, all nested in *Helichrysum*. In the ML tree inferred from ITS (**Supplementary Figure S2**), *Anaphalis* was monophyletic, but in the other trees constructed by nrDNA, *Anaphalis* was polyphyletic (**Figure 2** and **Supplementary Figures S3**-**S6**). Four clades were included in *Anaphalis* with strong support. The topological relationships among the four clades were different in different phylogenetic trees. For example, in the ML tree based on ETS (**Supplementary Figure S4**), Clade 2 was sister to some *Helichrysum* taxa and the remaining *Anaphalis* taxa, and Clade 1 of *Anaphalis* was sister to Clade 3 and Clade 4. However, in the BI tree based on ETS (**Supplementary Figure S5**), Clade 1 was sister to some *Helichrysum* taxa and the remaining *Anaphalis* taxa; Clade 2 was sister to *Helichrysum*; and Clade 3 and Clade 4 were clusted together. Among the four clades, Clade 2 included only two species: *A. subdecurrens* and *A. brevifolia.* There were 20, 11, and 36 taxa included in Clade 1, Clade 2, and Clade 4, respectively. Additionally, the samples of two species, *A. contorta* Hook.f. and *A. nepalensis* (Spreng.) Hand.-Mazz., in *Anaphalis* were not clustered into the same main clade (Clade 1 and Clade 3, respectively).

In *Anaphalis*, the species in Clade 1 + 3 (cp genome tree, **Figure 1A**) were same as those in Clade 1 and Clade 3 (nrDNA tree, **Figure 1B**), and the species in Clade 4 of cp genome tree (**Figure 1A**) were same as those in Clade 4 of nrDNA tree (**Figure 1B**). In the cp genome tree, *A. subdecurrens* was nested in *Pseudognaphalium*, and *A. brevifolia* was sister to *Helichrysum* (**Figure 1A**). However, in the nrDNA tree, these two species were clustered together (Clade 2) and sister to *Helichrysum* (**Figures 1B**, **2**).

### 3.2 Divergence time estimation and diversification through time

**Figure 3:**
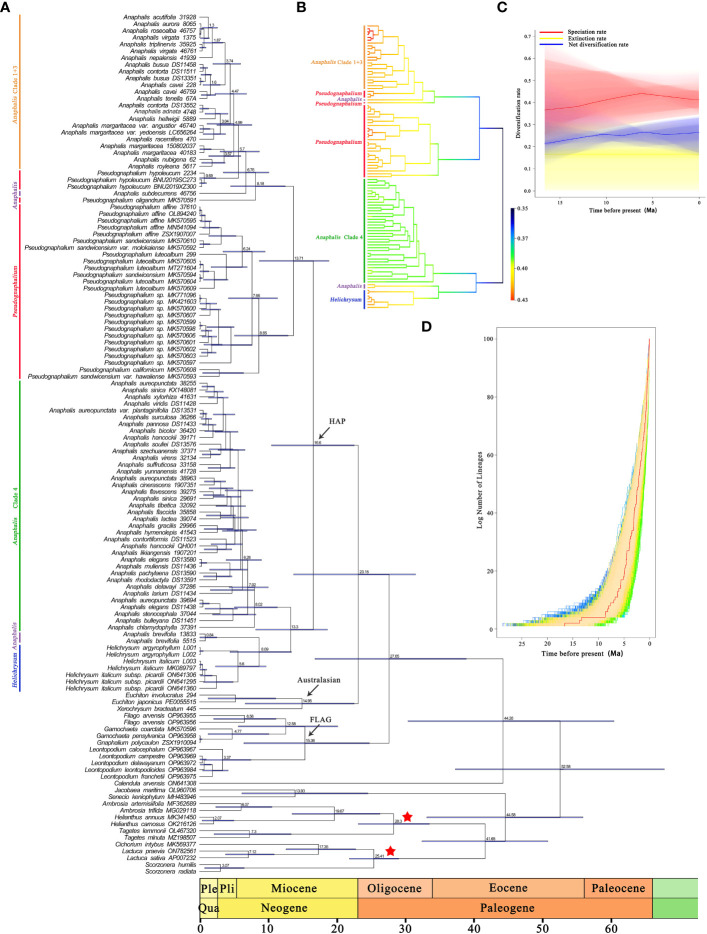
Divergence times estimation and BAMM analysis of HAP clade based on complete plastome sequences. **(A)** Time-calibrated phylogenetic tree of the HAP clade and its closely related genera, inferred with BEAST from the 127 represent samples plastid dataset. Bars on the nodes indicate the 95% HPD intervals, numbers on the bars indicate the mean age (Mya). The calibrated nodes are indicated by red asterisk. **(B)** Speciation rate dynamics of HAP clade estimated by BAMM. Branch color reflects the mean of the marginal posterior density of speciation rates for each segment of the branches, with rates increasing from blue to red. **(C)** The diversification rates from the origin of the HAP clade to the present obtained using BAMM. **(D)** LTT plots show the cumulative number of lineages over time of HAP clade. The red line shows the maximum clade credibility tree.

In the cpDNA date analysis (**Figure 3A** and **Supplementary Figure S7**), the ancestor of HAP clade diverged approximately 16.6 Mya (95% HPD: 10.49–22.57 Mya). In HAP clade, the MRCA of *Helichrysum*, *A. brevifolia*, and Clade 4 of *Anaphalis* diverged at 13.3 Mya (95% HPD: 8.2–18.66 Mya). Divergence between *A. brevifolia* and its sister genera, *Helichrysum*, was estimated to have occurred during the Miocene, at 8.69 Mya (95% HPD: 4.44–13.4 Mya). The MRCA of Clade 4 in *Anaphalis* diverged at 8.02 Mya (95% HPD: 5.09–11.36 Mya). Divergence among *Pseudognaphalium*, *A. subdecurrens*, and Clade 1 + 3 of *Anaphalis* was estimated to also have occurred during the Miocene, at 13.71 Mya (95% HPD: 8.72–18.88 Mya). The MRCA of most *Pseudognaphalium* species diverged at 8.65 Mya (95% HPD: 5.11–12.92 Mya). The ancestor of Clade 1 + 3 in *Anaphalis* diverged approximately 4.99 Mya (95% HPD: 2.53–7.64 Mya) during the Pliocene.

The BAMM analysis indicated that no apparent rate shift occurred in the phylogeny of the HAP clade (**Figure 3B**). The radiation of the HAP species began in the Miocene (ca. 16.6 Mya) at a rate of 0.35 lineages per million years. This analysis indicated that the HAP species diversification was characterized by an early relatively lower initial speciation rate, followed by a burst of speciation. Additionally, *Pseudognaphalium*, *Helichrysum*, and Clade 1 + 3 of *Anaphalis* had higher speciation rates than that of Clade 4 of *Anaphalis*. The rate-through-time plot shows that the speciation rate in the HAP clade gradually increased before ca. 6 million years, and then slightly declined (**Figure 3C**). The extinction rate rose in fluctuation. However, the net diversification rate in the HAP clade was constant at a low level. The semi-logarithmic LTT plot analysis corroborated the results of BAMM analysis and indicated an accelerated lineage accumulation after ca. 8 million years (**Figure 3D**).

### 3.3 Ancestral geographical range

**Figure 4:**
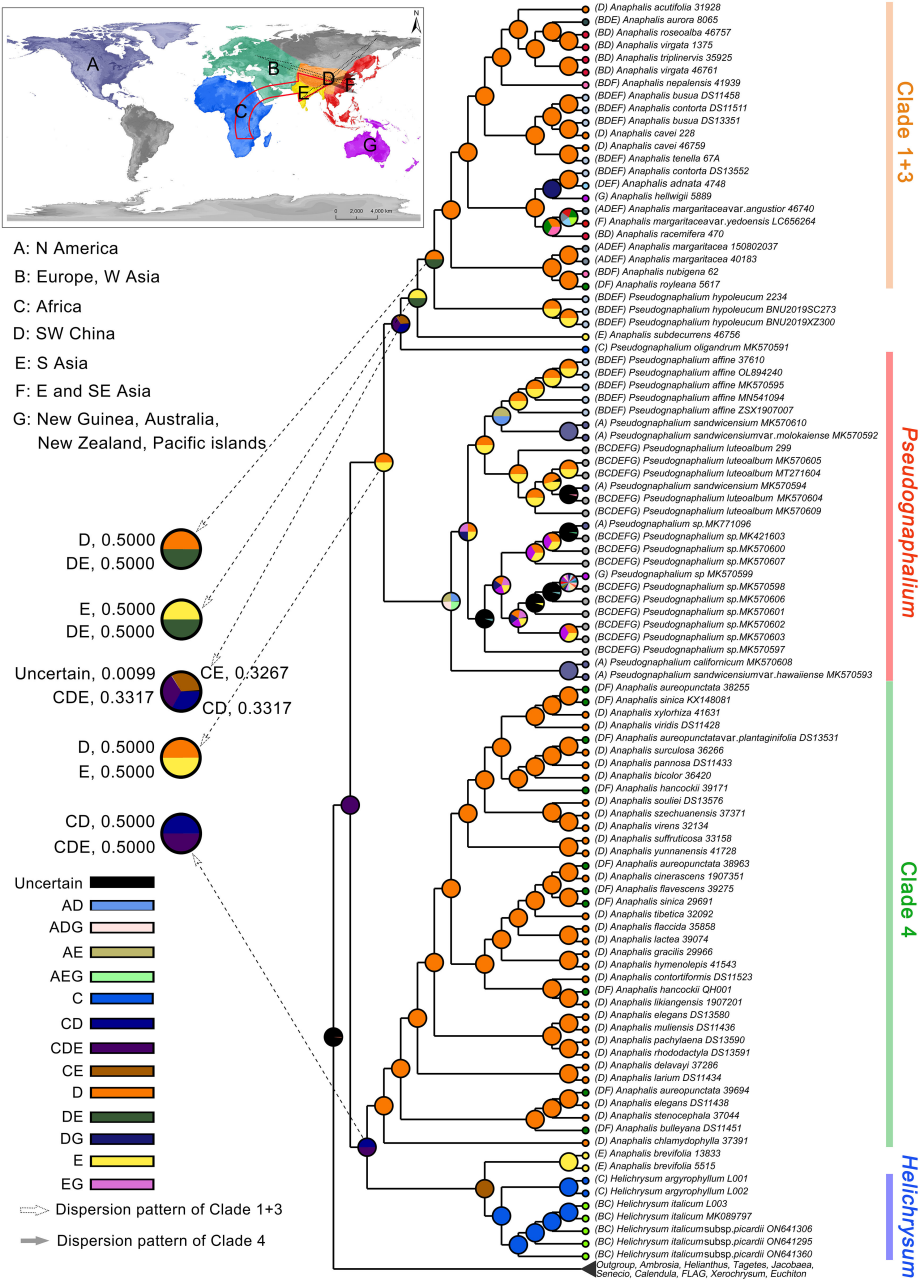
Ancestral area reconstruction of the HAP clade under the S-DIVA model in RASP v4.2 and using the phylogenetic tree from the BEAST analysis. Pie charts denote the ancestral areas with probability values. The map shows the coding areas in different colors.

Based on cp genomes, the ancestral area reconstruction using S-DIVA model inferred that the ancestor of HAP clade probably occurred in Africa, Southern Asia, and/or Southwestern China, (coding: CDE) (**Figure 4**). The MRCA of *Helichrysum*, *A. brevifolia*, and Clade 4 of *Anaphalis* remained in CD (Africa, Southwestern China), and/or CDE (probability = 0.5000, respectively) areas. The ancestor of *Helichrysum* and *A. brevifolia* probably occurred in Africa and/or Southern Asia (coding: CE). The ancestor of *A. brevifolia* occurred in Southern Asia (coding: E). The MRCA of Clade 4 of *Anaphalis* remained in Southwestern China (coding: D). The ancestor of *Pseudognaphalium*, *A. subdecurrens*, and Clade 1 + 3 of *Anaphalis* remained in the D and/or E (probability = 0.5000, respectively) areas. The MRCA of *P. oligandrum*, *P. hypoleucum*, *A. subdecurrens*, and Clade 1 + 3 of *Anaphalis* remained in the CD, CE, and/or CDE (probability = 0.3317, 0.3267, and 0.3317, respectively) areas. Then, except for *P. oligandrum*, the ancestor of taxa mentioned above probably occurred in the E and/or DE (probability = 0.5000, respectively) areas. The MRCA of *P. hypoleucum* and its sister clade, Clade 1 + 3 of *Anaphalis*, remained in the D and/or DE (probability = 0.5000, respectively) areas. The ancestor of Clade 1 + 3 of *Anaphalis* occurred in the D area.

### 3.4 The ancestral state reconstruction of morphological characters

**Figure 5:**
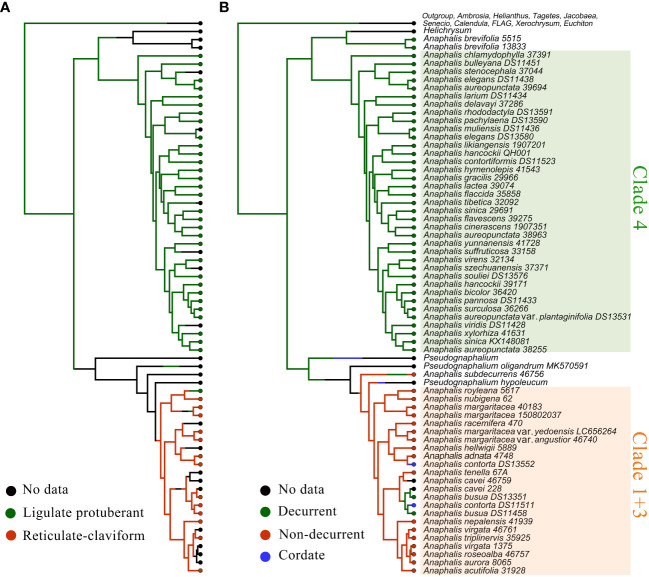
The ancestral state reconstruction and stochastic character mapping of morphological characters performed in R v4.1, using the make.simmap commands under ER model in phytools package. **(A)** Achene surface ornamentation **(B)** Leaf base.

The characters of achene surface ornamentation and leaf base and the reconstruction of these characters are presented in **Figure 5**. In *Anaphalis*, the taxa in Clade 4 had ligulate protuberant surface ornamentation of achene; in Clade 1 + 3, the taxa had reticulate-claviform surface ornamentation of achene, except *A. royleana* DC. (**Figure 5**). The ancestral state of achene surface ornamentation in *Anaphalis* was probably ligulate protuberant. And, the reticulate-claviform surface ornamentation of achene might evolve from the ligulate protuberant surface ornamentation. The leaf bases of *A. brevifolia* and the taxa in Clade 4 were decurrent. Most taxa in Clade 1 + 3 and *A. subdecurrens* had non-decurrent leaf bases. In Clade 1 + 3, *A. contorta* had a cordate leaf base, and *A. busua* DC. had a decurrent leaf base. The ancestral state of leaf base in *Anaphalis* was probably decurrent, and the cordate and non-decurrent leaf base might evolve from the decurrent leaf base.

## 4 Discussion

### 4.1 Topological discordance between the nrDNA and cpDNA trees

In this study, the topological trees constructed based on complete cp genomes (**Figure 1A** and **Supplementary Figure S1**) and nrDNA (**Figures 1B**, **2** and **Supplementary Figures S2**–**S6**) were incongruent. Various hypotheses have been proposed in previous studies to explain this phenomenon, such as ancient rapid radiation, incomplete lineage sorting, hybridization, introgression, and undersampling (Joly et al., 2009; Lo and Donoghue, 2012; Liu et al., 2017; Xu et al., 2017; Liu et al., 2020; Duan et al., 2021). These complex phylogenetic relationships are commonly found in Asteraceae (Bayer et al., 2002; Hidalgo et al., 2006; Kim et al., 2007; Barker et al., 2009; Galbany-Casals et al., 2010; Zhao et al., 2010; Jara-Arancio et al., 2017). The taxa in Clade 1 and Clade 3 of *Anaphalis* (nrDNA trees) were same as those in Clade 1 + 3 (cp genome trees), which suggested that the cp genetic information of these taxa was similar. However, in the nrDNA trees, Clade 3 was closely related to Clade 4 and distant from Clade 1, and the samples of *A. contorta* and *A. nepalensis* were clustered into Clade 1 and Clade 3, respectively. In previous studies, the past or present hybrid events were used to interpret the discordant phylogenetic relationships of several genera in tribe Gnaphalieae, rather than the sorting or retention of ancestral polymorphisms (Smissen et al., 2004; Smissen and Breitwieser, 2008; Galbany-Casals et al., 2010; Smissen et al., 2011). Furthermore, Smissen et al. (2011) hypothesized that *Anaphalis* and the Mediterranean-Asian *Helichrysum* had a common allopolyploid origin with possible parental species shared based on additional evidence of low-copy markers and chromosome data. However, only one *Anaphalis* species was represented in their study, *A. margaritacea*. Meng et al., 2010; Meng et al., 2014) reported the chromosome numbers of 18 *Anaphalis* taxa and indicated that tetraploidy is common in *Anaphalis* (the detailed information are shown on **Figure 1**), which further supported the polyploidy origin of the genus.

In the present study, we inferred that allopolyploid origin (or evolving from a polymorphic polyploid ancestral complex) and hybridization probably all occurred in *Anaphalis*. First, we hypothesized that there were two lineages of *Anaphalis*, Clade 1 + 3 and Clade 4 (excluding *A. subdecurrens* and *A. brevifolia*), based on our cpDNA analysis. The species in Clade 1 + 3 (identical to Clade 1 and Clade 3) and Clade 4 possibly evolved from *Pseudognaphalium* and *Helichrysum*, respectively, based on our cpDNA phylogenetic trees. Alternatively, the main clades of *Anaphalis* evolved from a polymorphic polyploid ancestral complex of *Helichrysum*. Then, the species in Clade 3 might have hybridized with those in Clade 4, leading to these two clades being closely related. However, this hypothesis lacked sufficient support because Clade 3 was sister to Clade 4 with strong support values instead of clustering together. The other hypothesis was that there were three lineages of *Anaphalis* (excluding *A. subdecurrens* and *A. brevifolia*) based on our nrDNA analysis, which possibly evolved from *Helichrysum* respectively. Then, the taxa in Clade 3 might have hybridized with those in Clade 1 or captured the cp from Clade 1, resulting in those taxa clustered into one clade (Clade 1 + 3) in the cpDNA phylogenetic trees. Chloroplast capture, a stochastic process that occurs in groups evolving through rapid radiation (the Asteraceae family is highly evolved and is in the stage of rapid differentiation), can influence cp genome (Rieseberg and Soltis, 1991; Bremer, 1994; Fior et al., 2013). Nevertheless, in the nrDNA tree, the samples of *A. contorta* and *A. nepalensis* were not clustered into the same main clade but rather clustered into Clade 1 and Clade 3, respectively, potentially reducing support for our hypothesis described above. Other possible reasons also could be used to explain the discordant phylogenetic relationships between nrDNA and cpDNA, such as nrDNA polymorphism, low sequence resolution, or incomplete lineage sorting, which were commonly discussed in Asteraceae (Álvarez and Wendel, 2003; Galbany-Casals et al., 2004; Ford et al., 2007; Smissen and Breitwieser, 2008; Galbany-Casals et al., 2011; Güzel et al., 2021; Thode et al., 2021). Although the exact reasons that led to these complex phylogenetic relationships are not certain with the present data, the systematic position of Clade 4 was clear. Additionally, it seems probable that more samples of *Helichrysum* and *Pseudognaphalium* are required to discover the complex phylogenetic relationships and histories.

### 4.2 Phylogenetic relationships within *Anaphalis*

Based on the current most comprehensive sampling of *Anaphalis*, both our phylogenetic analyses using nrDNA and cp genomes did not support the monophyly of this genus. The phylogenetic analyses based on nrDNA (ITS and ETS) by Nie et al. (2013) focusing on *Anaphalis* indicated that the monophyly of *Anaphalis* was weakly supported. Galbany-Casals et al. (2014) explored the phylogenetic relationships in *Helichrysum* and related genera, and seven *Anaphalis* taxa were included in their analyses. Similarly, the phylogenetic tree based on nrDNA also weakly supported the monophyly of *Anaphalis*. However, in their phylogenetic tree inferred using cpDNA markers (*rpl32-trnL* and *ndhF*), *Anaphalis* was not monophyletic and included two clades, both nested within *Helichrysum* (Galbany-Casals et al., 2014).

Additionally, our phylogenetic analysis results did not support the traditional infrageneric taxonomy system of *Anaphalis*. Based on the size of capitulum, Hooker (1882) divided *Anaphalis* into two series: series I the diameter of capitulum ca. 1/2–2/3 inch; series II, the diameter of capitulum ca. 1/6–1/3 inch. Nevertheless, our phylogenetic results did not support the monophyly of these series. For example, *A. nubigena*, *A. royleana*, *A. triplinervis*, and *A. xylorhiza* belonged to series I (Hooker, 1882), but in our phylogenetic trees, *A. xylorhiza* did not cluster together with the other three species (**Figure 1**). Borissova (1999) proposed two sections (Sect. *Anaphalis* and Sect. *Polycephales*) based on the number and the size of capitula. Sect. *Anaphalis* with fewer and larger capitula included two species, *A. serawschanica* (Winkl.) B. Fedtsch. growing on the mountain slopes of Central Asia and *A. nubigena* in the alpine zone of the Himalayas. Sect. *Polycephales* were distributed across Asia, particularly in the Himalayas, and included *A. margaritacea*, *A. racemifera*, *A. roseoalba*, *A. virgata*, etc. Obviously, *A. nubigena* was closely related to species of Sect. *Polycephales* in our phylogenetic analyses (**Figure 1**). Chen et al. (1966) focused on Chinese *Anaphalis* species and presented an infrageneric taxonomy system that included two subgenera (Subgen. *Gnaphaliops* and Subgen. *Anaphalis*), two sections, and ten series. However, the results of our phylogenetic analyses suggested that none of these were monophyletic. The Subgen. *Gnaphaliops* included only one species, *A. bulleyana* (Chen et al., 1966), but this species was not obviously separated from other *Anaphalis* species in our phylogenetic analyses. In the phylogenetic tree inferred by complete cp genomes (**Figure 1A**), *A. bulleyana* was clustered with *A. aureopunctata*, *A. elegans*, and *A. stenocephala*. In the nrDNA tree, *A. bulleyana* was closely related to *A. aureopunctata*, *A. sinica*, *and A. delavayi* (**Figure 2**). Two sections, Sect. *Margaripes* and Sect. *Anaphalis*, were included in Subgen. *Anaphalis*, mainly based on the number, size, and shape of capitula (Chen et al., 1966). Whereas, the results of our phylogenetic analysis supported that these two sections were nested with each other. For instance, *A. contorta*, *A. margaritacea*, and *A. oxyphylla* belonged to Sect. *Margaripes*; and *A. acutifolia*, *A. nepalensis*, and *A. triplinervis* belonged to Sect. *Anaphalis* (Chen et al., 1966). Nevertheless, these species were clustered into one clade in our phylogenetic analyses: Clade 1 + 3 of the cpDNA tree (**Figure 1A**) and Clade 1 of the nrDNA tree (**Figure 1B**). The same problem existed in the series proposed by Chen et al. (1966).

Recent phylogenetic analyses based on two nrDNA markers (ITS and ETS) supported two major lineages of *Anaphalis* that corresponded to the shape of the leaf base rather than the characters of the capitula (Nie et al., 2013). These two lineages were the “non-decurrent” clade (species with attenuate or narrowed leaf bases but never forming wings on the stem) and the “decurrent” clade (most species with decurrent leaf base and a small group with cordate leaf base). The main clades inferred by Nie et al. (2013) were mostly consistent with those of our nrDNA tree (**Figure 1B**). Species of the “non-decurrent” clade (Clade I and Clade II; Nie et al., 2013) were all included in Clade 1 of our nrDNA tree (**Figure 1B).** Three species, *A. cntorta*, *A. plicata*, and *A. hondae*, with cordate leaf bases were clustered with *A. busua* with decurrent leaf base, forming a single main clade (Clade III), which was sister to other species (Clade IV) with decurrent leaf bases (Nie et al., 2013). However, as more samples were included in our nrDNA tree, we found that the species with cordate leaf bases (*A. contorta*, *A. hondae*, and *A. plicata*), decurrent leaf bases (*A. busua* and *A. nagasawai*), and non-decurrent leaf bases (*A. cavei*, *A. nepalensis*, *A. javanica*, and *A. longifolia*) were all nested together and constituted one main clade (Clade 3), which was sister to other species (Clade 4) with decurrent leaf bases (**Figure 1B**). Additionally, our cpDNA phylogenetic tree also suggested that these species with different leaf base were nested (**Figure 5B**, Clade 1 + 3). As a result, the character of leaf base did not perfectly support these phylogenetic relationships. Nevertheless, compared with the characters of capitula, the genetic data suggested that the shape of leaf base appeared to show less homoplasy and was largely congruent with the phylogenetic relationships. Therefore, the significance of leaf base characteristics for infrageneric taxonomy system of *Anaphalis* should not be ignored. According to the characteristics of achene micro-morphology, Xu et al. (2021) divided 39 Chinese *Anaphalis* taxa into two groups: Group I with reticulate-claviform surface ornamentation and Group II with ligulate protuberant surface ornamentation. We compared the taxa in the two groups with those in the main clades of our phylogenetic trees. Then, we found that the taxa in Group I were all included in Clade 1 + 3 of cpDNA tree and in Clade 1 and Clade 3 of nrDNA tree. However, the taxa in Group II were divided into different main clades of our phylogenetic trees (cpDNA tree, Clade 1 + 3 and Clade 4; nrDNA tree, Clade 1 and Clade 4), because *A. royleana* had a different achene micro-morphology from other taxa in the same clade. Thus, similar to leaf base, although the characteristics of achene micro-morphology also did not completely support the phylogenetic relationships of *Anaphalis*, they are still important for the infrageneric taxonomy system of this genus because of their less homoplasy.

Therefore, compared with nrDNA trees, the cp trees were more effective for phylogenetic resolution and were supported by the morphological (achene surface ornamentation and leaf base) characters. The morphological characters evolution analysis indicated that ligulate protuberant surface ornamentation on achene was a morphological synapomorphy of Clade 4. In Clade 1 + 3, except *A. royleana*, the other taxa had reticulate-claviform surface ornamentation on achene. Chen and Jing (2007) inferred reticulate-claviform ornamentation was the more primitive type. However, based on our analyses, the ligulate protuberant surface ornamentation was the ancestral state of *Anaphalis* and evolved once in Clade 1 + 3 (**Figure 5A**). The presence of decurrent leaf base was a morphological synapomorphy of Clade 4. Most species of Clade 1 + 3 had non-decurrent leaf bases, and a few species had decurrent or cordate leaf bases. Decurrent leaf base was the ancestral state of *Anaphalis*. The characters of decurrent and cordate leaf base evolved twice in Clade 1 + 3, respectively (**Figure 5B**). The phylogenetically conserved pattern of leaf base in Clade 4 might be due to greater genetic constraints and/or stabilizing selection pressure favoring stasis of this character in alpine habitats, because most species of this clade were restricted to the Himalayan region. Recent phylogenetic analyses of *Anaphalis* (emphasis on the eastern Himalayan taxa) and *Leontopodium* (diversified in Himalayas and Hengduan Mountains) also suggested a higher degree of homoplasy in leaf base (Nie et al., 2013; Xu et al., 2023b).

Compared with the infrageneric taxonomy system, the interspecies relationships of *Anaphalis* were more complex. It was common for samples from the same species not to cluster together, such as *A. aureopunctata*, *A. busua*, *A. contorta*, and *A. margaritacea* (**Figures 1A**, **2**). Furthermore, the inconsistent phylogenetic results inferred from different DNA data also reflected the complexity of interspecific relationships in *Anaphalis*. For instance, in the cpDNA phylogenetic tree, *A. bulleyana* was closely related to *A. aureopunctata*, *A. elegans*, and *A. stenocephala* (**Figure 1A**), whereas in the nrDNA tree, this species was closely related to *A. aureopunctata* and *A. surculosa* (**Figure 2**). Frequent hybridization, introgression, horizontal gene transfer, polyploidy accompanied by apomixis, or rapid radiation may all contribute to the evolutionary complexity (Zou and Ge, 2008; Liu et al., 2020). Particularly in Asteraceae, which is highly evolved and in the rapid differentiation stage, a substantial number of complex and polymorphic transitional taxa lead to several challenges in classification and phylogeny research (Bremer, 1994; Bayer et al., 2002; Hidalgo et al., 2006; Kim et al., 2007; Barker et al, 2009; Galbany-Casals et al., 2010; Zhao et al., 2010; Jara-Arancio et al., 2017). Recently, Xu et al. (2023b) indicated that the relationships within *Leontopodium* (Asteraceae, Gnaphalieae) were very complex and suggested that hybridization may be responsible for this phenomenon.

In *Anaphalis*, particular attention should be paid to three species: *A. adnata* Wall. ex DC., *A. subdecurrens*, and *A. brevifolia*. De Candolle (1837) first described *A. adnata*. Then, this species was transferred to *Gnaphalium*, as *G. adnatum* (DC.) Wall. ex Thwaites (Thwaites, 1864), which was also recognized by Ling (1979). In the *Flora of China*, this species was included in *Pseudognaphalium*, as *P. adnatum* (DC.) Y. S. Chen (Chen et al., 2011). Clearly, our phylogenetic results indicated that this species should be included in *Anaphalis* (**Figures 1**, **2**). Furthermore, the characters of achene surface ornamentation and leaf base of this species were same as the taxa in Clade 1 + 3 of *Anaphalis* (**Figure 5**). Therefore, we suggest that *P. adnatum* should be considered as a synonym of *A. adnata.* Our phylogenetic trees showed that *A. subdecurrens* and *A. brevifolia*, which were distributed in South India and Sri Lanka, had a close relationship with *Pseudognaphalium* and *Helichrysum* (**Figure 1**). Subdioecy was widely used to distinguish *Anaphalis* from its related genera, such as *Pseudognaphalium*, *Helichrysum*, and *Gnaphalium* (Bentham and Hooker, 1873; Chen et al., 1966; Drury, 1970; Ling, 1979; Anderberg, 1991). However, Grierson (1972) clarified that the *Anaphalis* species in Sri Lanka were not subdioecious. Thus, our phylogenetic results support the use of subdioecy to distinguish *Anaphalis* from its related genera and the transfer of *A. subdecurrens* and *A. brevifolia* to *Pseudognaphalium* or *Helichrysum*. Nevertheless, because we did not conduct a sufficiently detailed study and investigation of the characters of these two species, it may be too early to draw such revises.

### 4.3 Historical biogeography

Based on cp genome data, the crown ages of the HAP clade were estimated to be 16.6 (10.49–22.57) Mya, suggesting that its early diversification occurred in the Miocene, and the two main clades of HAP diverged during the middle Miocene (**Figure 3A**). Based on nrDNA data, Nie et al. (2016) focusing on Gnaphalieae estimated the crown ages of HAP clade at to be approximately 15.39 (11.42–19.64) Mya, which was similar to our estimate. Our results also revealed that the three genera, *Anaphalis*, *Pseudognaphalium*, and *Helichrysum*, diverged in the late Miocene (**Figure 3A**). The Miocene was a pivotal period in the “making of the modern world” owing to specific climatic conditions (such as the overall cooling trend of the climate, the origin of modern ocean currents, and the aridification of continental interiors) and geological events (such as the uplift of the Himalayan mountains, the closure of the Tethys Ocean, and the closing of the Inter-American Seaway). This resulted in it being a key period for angiosperm phylogeny, marked by pronounced rates of dispersal (Zachos et al., 2008; Antonelli, 2009; Clark et al., 2009; Potter and Szatmari, 2009; Couvreur et al., 2011; Bacon et al., 2012; Yu et al., 2014; Nie et al., 2016; Huang et al., 2018; Dong et al., 2022). The crown age of the clade including *Helichrysum* and *A. brevifolia* was estimated to be 8.69 (4.44–13.4) Mya (**Figure 3A**). The crown age of the clade including *P. oligandrum*, *P. hypoleucum*, *A. subdecurrens*, and Clade 1 + 3 of *Anaphalis* was estimated to be 8.18 (4.53–12.5) Mya (**Figure 3A**). The crown age of the remaining *Pseudognaphalium* species was estimated to be 8.65 (5.11–12.92) Mya (**Figure 3A**). The crown ages of Clade 1 + 3 and Clade 4 of *Anaphalis* were estimated to be 4.99 (2.53–7.64) Mya and 8.02 (5.09–11.36) Mya, respectively (**Figure 3A**). These clades suggested that HAP clade underwent rapid diversification in the late Miocene to Pliocene. Our LTT plot analysis indicated an accelerated lineage accumulation after ca. 8 Mya (**Figure 3D**). This acceleration may be attributed to the dry habitat during late Miocene to Pliocene, providing favorable open grassland for dispersion (Palazzesi and Barreda, 2012; Nie et al., 2016). Similar cases have been found in many other flowering plants (Coleman et al., 2003; Li et al., 2009; Thiv et al., 2010; Nie et al., 2016; Pouchon et al., 2023), indicating the importance of the late Miocene to Pliocene for the assembly of modern flora. Additionally, our BAMM analysis results revealed that the HAP species experienced an early relatively lower speciation rate (middle Miocene) followed by a burst of diversification (from late Miocene to Pliocene), with a relatively constant extinction rate (**Figures 3B, C**). We estimated diversification rates beginning from ca. 16.6 Mya, which had a rate of 0.35 species per million years. This reported rate was similar to those found in other taxa, such as *Saxifraga* Tourn. ex L., *Gentiana* (Tourn.) L., and *Delphinium* L. (Jabbour and Renner, 2012; Favre et al., 2016; Ebersbach et al., 2017). Although the diversification rate of the HAP clade was not low compared with other angiosperm clades (Xing and Ree, 2017; Qian et al., 2018), it did not match the exceptionally high rate recorded in another taxa of Asteraceae, the *Espeletia* Mutis ex Bonpl. complex, which most likely caused by the Pleistocene climatic oscillations that triggered exceptionally rapid radiation (Pouchon et al., 2018). Regardless, our data added another empirical example of the rapid differentiation of Asteraceae plants, further demonstrating that high diversification rates occurred from the late Miocene to Pliocene.

Based on cp genomes, we were unable to determine the specific origin area of the HAP clade, likely due to the limited inclusion of *Helichrysum* samples. Using nrDNA and cpDNA markers and focusing on *Helichrysum*, Galbany-Casals et al. (2014) suggested that the HAP clade originated in the Cape region of Southern Africa and subsequently dispersed to and diversified in the Eastern and Southern Africa. The biogeographic analyses of Nie et al. (2016) and Andrés-Sánchez et al. (2019) also indicated a Southern African origin for the HAP clade. Our biogeographic analyses confirmed that the ancestor of *Helichrysum* occurred in Africa (**Figure 4**), which was consistent with the inference of Galbany-Casals et al., 2004; Galbany-Casals et al., 2014). *Pseudognaphalium* is a large genus with a world-wide distribution, likely resulting from recent global expansions during the late Miocene to Pliocene (Nie et al., 2016). Our results supported two separate lineages of *Pseudognaphalium*, one of which was nested with *Anaphalis* and showed an African origin, but the origin area of another independent lineage remained undefined (**Figure 4**). Two separate lineages of *Pseudognaphalium* were also inferred by Nie et al. (2016), but both nested within the Southern African HAP clade, indicating a Southern African origin. For more reliable results, future investigations should include additional taxa and molecular data of *Pseudognaphalium* to reveal the biogeographic pattern of this genus in the future. Obviously, the ancestral region of *Anaphalis* was definitively in Southwestern China, regardless of *A. subdecurrens* and *A. brevifolia*, but the dispersal patterns of the two *Anaphalis* lineages were markedly different (**Figure 4**). The two *Anaphalis* lineages appeared to have originated in Africa, then spread to Western and Southern Asia, and subsequently moved into Southwestern China and the pan-Himalayan region forming a diversity center (**Figure 4**). Then, Clade 1 + 3 of *Anaphalis* dispersed around the world, except in Africa and South America. The ancestral origin region and dispersal pattern of Clade 1 + 3 inferred in this study were mostly consistent with previous results. It was generally accepted that the extant Gnaphalieae originated in Southern Africa and then dispersed to other regions, often over long distances (Bergh and Linder, 2009; Ward et al., 2009; Nie et al., 2016). According to the current distribution area of *Anaphalis*, Galbany-Casals et al. (2014) inferred that this genus might have originated in East tropical Africa, radiated in the Himalayan range, further spread to Eastern Asia, and later reached North America, where only *A. margaritacea* is currently present, across the Bering Strait. Additionally, the chromosome numbers provided support for a common origin of *Anaphalis* and the Eurasian *Helichrysum* from an African ancestor (Nie et al., 2013). However, our results distinctly showed that the origin time and dispersion pattern of Clade 4 were different and unrelated to those of Clade 1 + 3. Clade 4 diverged from *Helichrysum* at 13.3 (8.2–18.66) Mya, whereas Clade 1 + 3 of *Anaphalis* diverged with *Pseudognaphalium* at 5.7 (3–8.73) Mya (**Figures 3**, **4** and **Supplementary Figure S7**). Moreover, the Clade 4 dispersed to Eastern and Southeastern Asia from the ancestral origin region. (**Figure 4**).

## 5 Conclusions

Our major results include: (1) *Achyrocline*, *Anaphalis*, *Galeomma*, *Pseudognaphalium*, and *Syncephalum* were nested with *Helichrysum* in the HAP clade. *Anaphalis* was polyphyletic and nested with *Helichrysum* and *Pseudognaphalium*. (2) Two and four mainly clades of *Anaphalis* were recognized in the cp genome and nrDNA phylogenetic trees, respectively. Compared with nrDNA trees, the cp trees were more effective for phylogenetic resolution, and were supported by the morphological and cp genome characters. (3) Subdioecy could be used to distinguish *Anaphalis* from its related genera. (4) The achene surface ornamentation and leaf base showed less homoplasy and supported the two lineages of *Anaphalis* inferred by cp genome. (5) The HAP clade underwent rapid diversification in the late Miocene to Pliocene. The crown ages of the two *Anaphalis* lineages were estimated at 4.99 (2.53–7.64) Mya and 8.02 (5.09–11.36) Mya, respectively. (6) The two *Anaphalis* lineages appeared to have originated in Africa, then spread to Western and Southern Asia, and subsequently moved into Southwestern China and the pan-Himalayan region forming a diversity center. The dispersal patterns of the two *Anaphalis* lineages were different. One dispersed around the world, except in Africa and South America. The other one dispersed to Eastern and Southeastern Asia from the ancestral origin region.

## Data availability statement

The datasets presented in this study can be found in online repositories. The names of the repository/repositories and accession number(s) can be found below: https://www.ncbi.nlm.nih.gov/genbank/ , OR727193-OR727268 https://www.ncbi.nlm.nih.gov/genbank/ , OR700107-OR700182 https://www.ncbi.nlm.nih.gov/genbank/ , OR700107-OR711342.

## Author contributions

XX: Conceptualization, Data curation, Formal analysis, Investigation, Methodology, Writing – original draft. HX: Investigation, Writing – original draft. ZY: Data curation, Formal analysis, Writing – original draft. ZW: Writing – review & editing. JG: Data curation, Investigation, Writing – original draft. DL: Data curation, Writing – review & editing. QL: Supervision, Writing – review & editing. SZ: Conceptualization, Funding acquisition, Investigation, Project administration, Resources, Writing – review & editing.

## Supplementary material

Supplementary Material 1 The alignment of chloroplast (cp) genome sequences.

Supplementary Material 2 The alignment of internal transcribed spacer (ITS) sequences.

Supplementary Material 3 The alignment of external transcribed spacer (ETS) sequences.

Supplementary Figure 1 Phylogenetic trees of the HAP clade and its closely related genera are inferred from the complete chloroplast genomes. **(A)** Topology of the ML tree. **(B)** Topology of the BI tree. **(C)** ML tree, with bootstrap values of ML and posterior probabilities of BI shown at each node. Bootstrap values higher than 70 and posterior probabilities higher than 0.90 are indicated on branches. “-” means that the bootstrap value/posterior probability is less than 70/0.90.

Supplementary Figure 2 Phylogenetic tree of the HAP clade and its closely related genera is inferred from ITS sequences using ML method. **(A)** Topologies. **(B)** The phylogenetic tree shows bootstrap values of ML at each node. Bootstrap values higher than 70 are indicated on branches.

Supplementary Figure 3 Phylogenetic tree of the HAP clade and its closely related genera is inferred from ITS sequences using BI method. **(A)** Topologies. **(B)** The phylogenetic tree shows posterior probabilities of BI at each node. Posterior probabilities higher than 0.90 are indicated on branches.

Supplementary Figure 4 Phylogenetic tree of the HAP clade and its closely related genera is inferred from ETS sequences using ML method. **(A)** Topologies. **(B)** The phylogenetic tree shows bootstrap values of ML at each node. Bootstrap values higher than 70 are indicated on branches.

Supplementary Figure 5 Phylogenetic tree of the HAP clade and its closely related genera is inferred from ETS sequences using BI method. **(A)** Topologies. **(B)** The phylogenetic tree shows posterior probabilities of BI at each node. Posterior probabilities higher than 0.90 are indicated on branches.

Supplementary Figure 6 Phylogenetic tree of the HAP clade and its closely related genera is inferred from the concatenated sequences of ITS and ETS using BI method. The posterior probabilities of BI are showed at each node. Posterior probabilities higher than 0.90 are indicated on branches.

Supplementary Figure 7 Time-calibrated phylogenetic tree of the HAP clade and its closely related genera, inferred with BEAST from the 127 represent samples plastid dataset. The numbers of 95% HPD intervals are showed at each node indicating the inferred age range (Mya).
