# Supplementary figures and images for: Phylogeny, biogeography, and character evolution of Anaphalis (Gnaphalieae, Asteraceae)
Source: Front Plant Sci. 2024 Feb 7;15:1336229. doi: 10.3389/fpls.2024.1336229 (PMC10879626; doi:10.3389/fpls.2024.1336229)

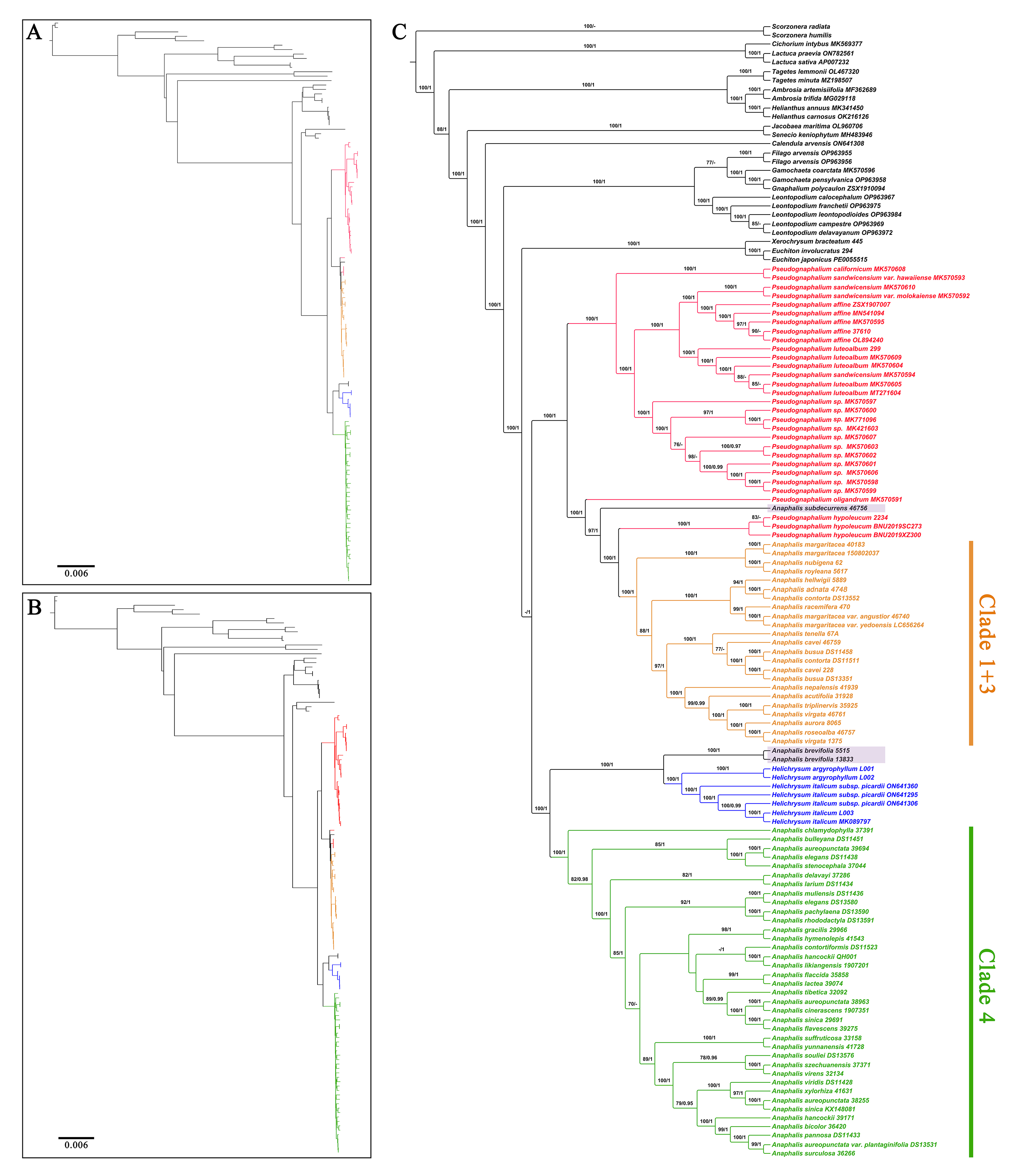

Supplement: Supplementary Figure 1 — Phylogenetic trees of the HAP clade and its closely related genera are inferred from the complete chloroplast genomes. (A) Topology of the ML tree. (B) Topology of the BI tree. (C) ML tree, with bootstrap values of ML and posterior probabilities of BI shown at each node. Bootstrap values higher than 70 and posterior probabilities higher than 0.90 are indicated on branches. “-” means that the bootstrap value/posterior probability is less than 70/0.90. [file Image_1.jpeg]

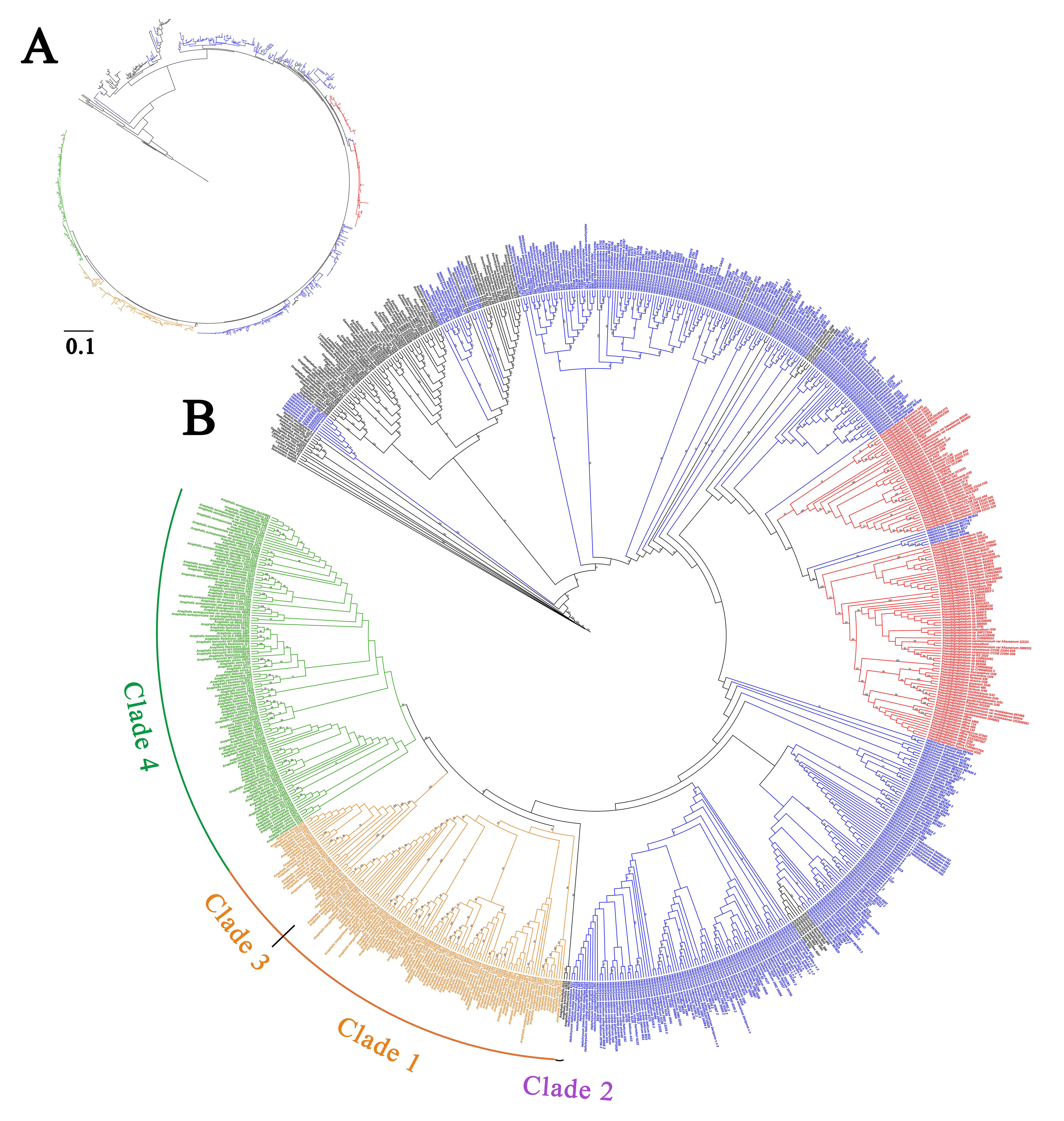

Supplement: Supplementary Figure 2 — Phylogenetic tree of the HAP clade and its closely related genera is inferred from ITS sequences using ML method. (A) Topologies. (B) The phylogenetic tree shows bootstrap values of ML at each node. Bootstrap values higher than 70 are indicated on branches. [file Image_2.jpeg]

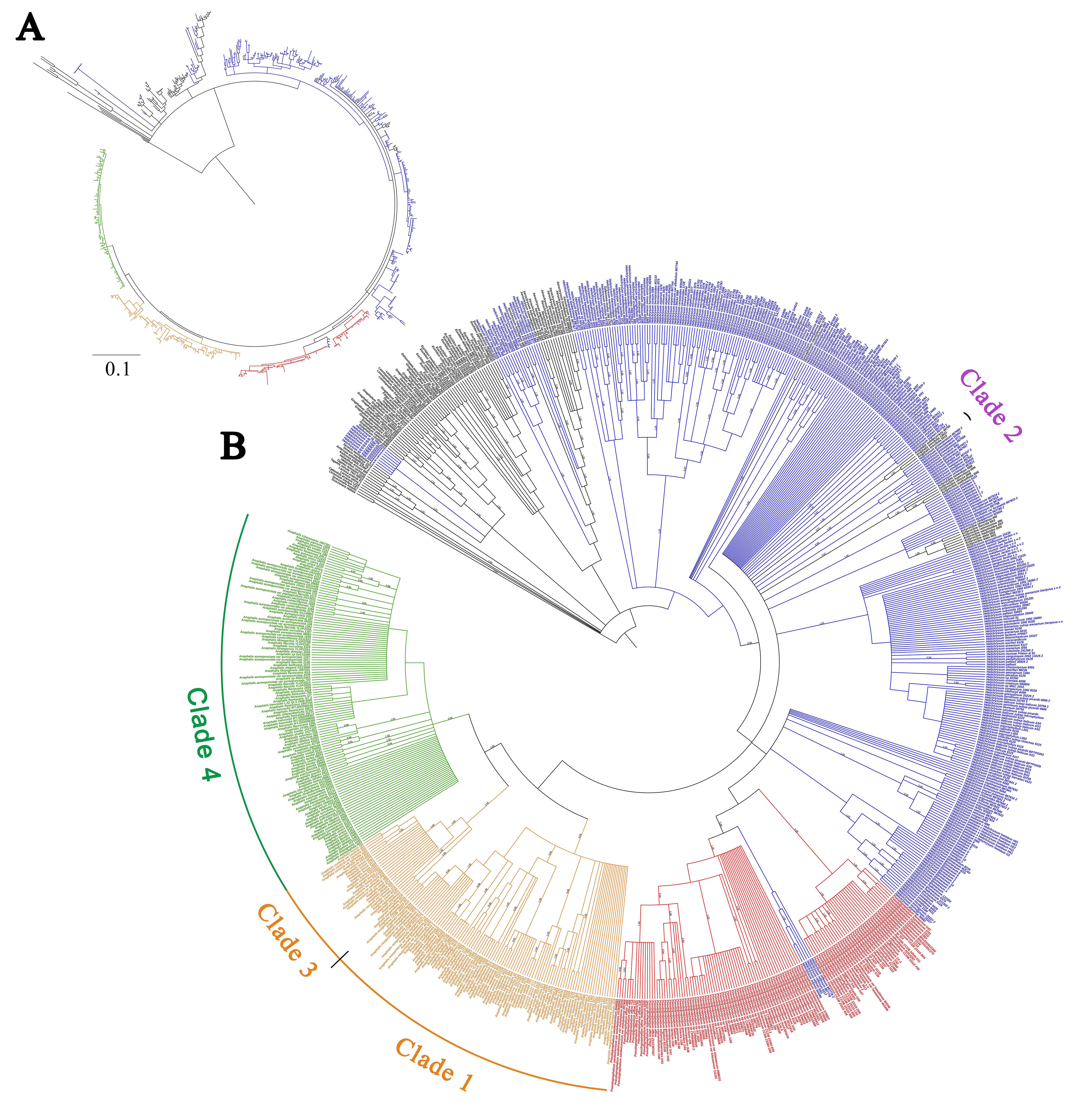

Supplement: Supplementary Figure 3 — Phylogenetic tree of the HAP clade and its closely related genera is inferred from ITS sequences using BI method. (A) Topologies. (B) The phylogenetic tree shows posterior probabilities of BI at each node. Posterior probabilities higher than 0.90 are indicated on branches. [file Image_3.jpeg]

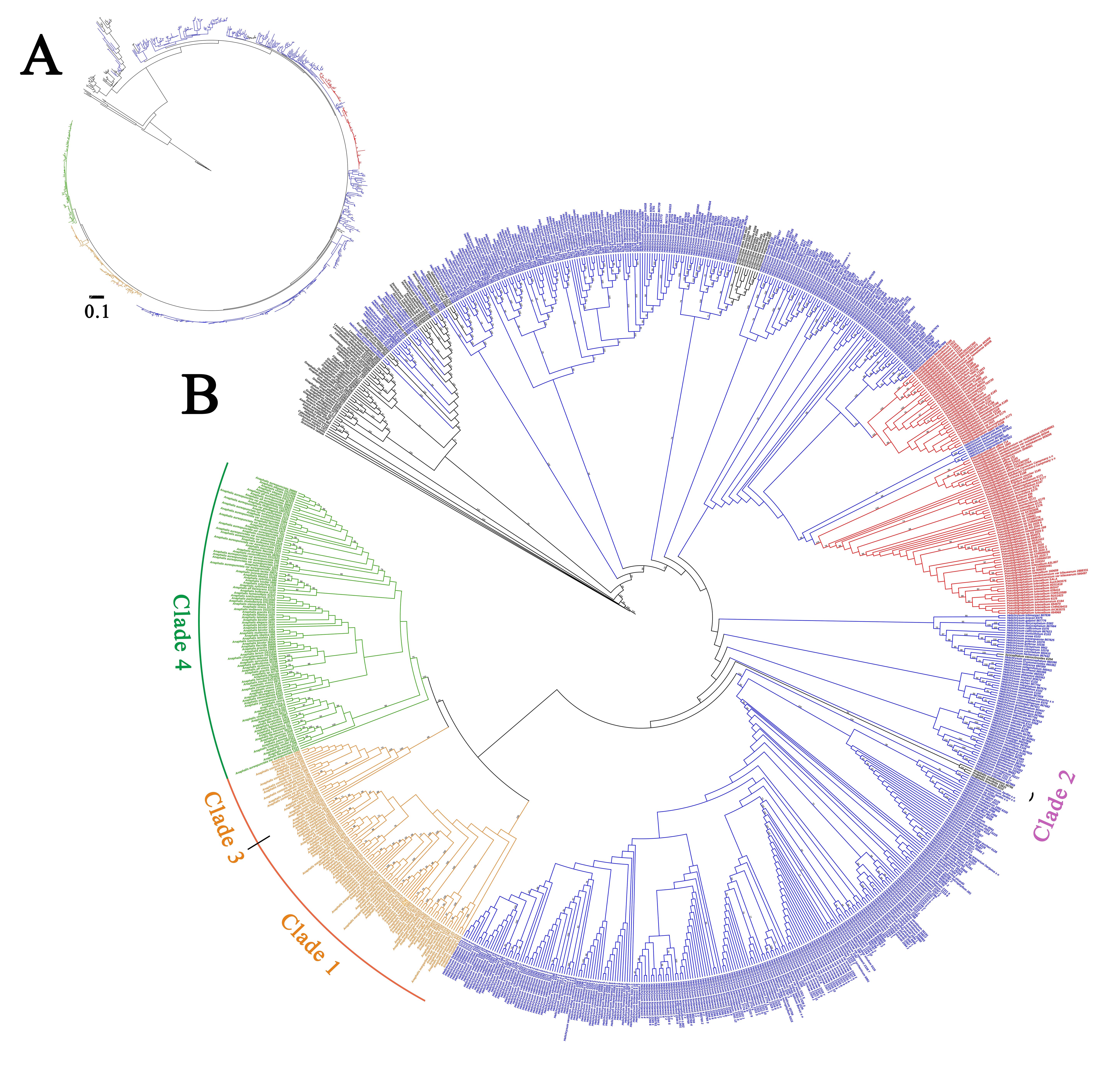

Supplement: Supplementary Figure 4 — Phylogenetic tree of the HAP clade and its closely related genera is inferred from ETS sequences using ML method. (A) Topologies. (B) The phylogenetic tree shows bootstrap values of ML at each node. Bootstrap values higher than 70 are indicated on branches. [file Image_4.jpeg]

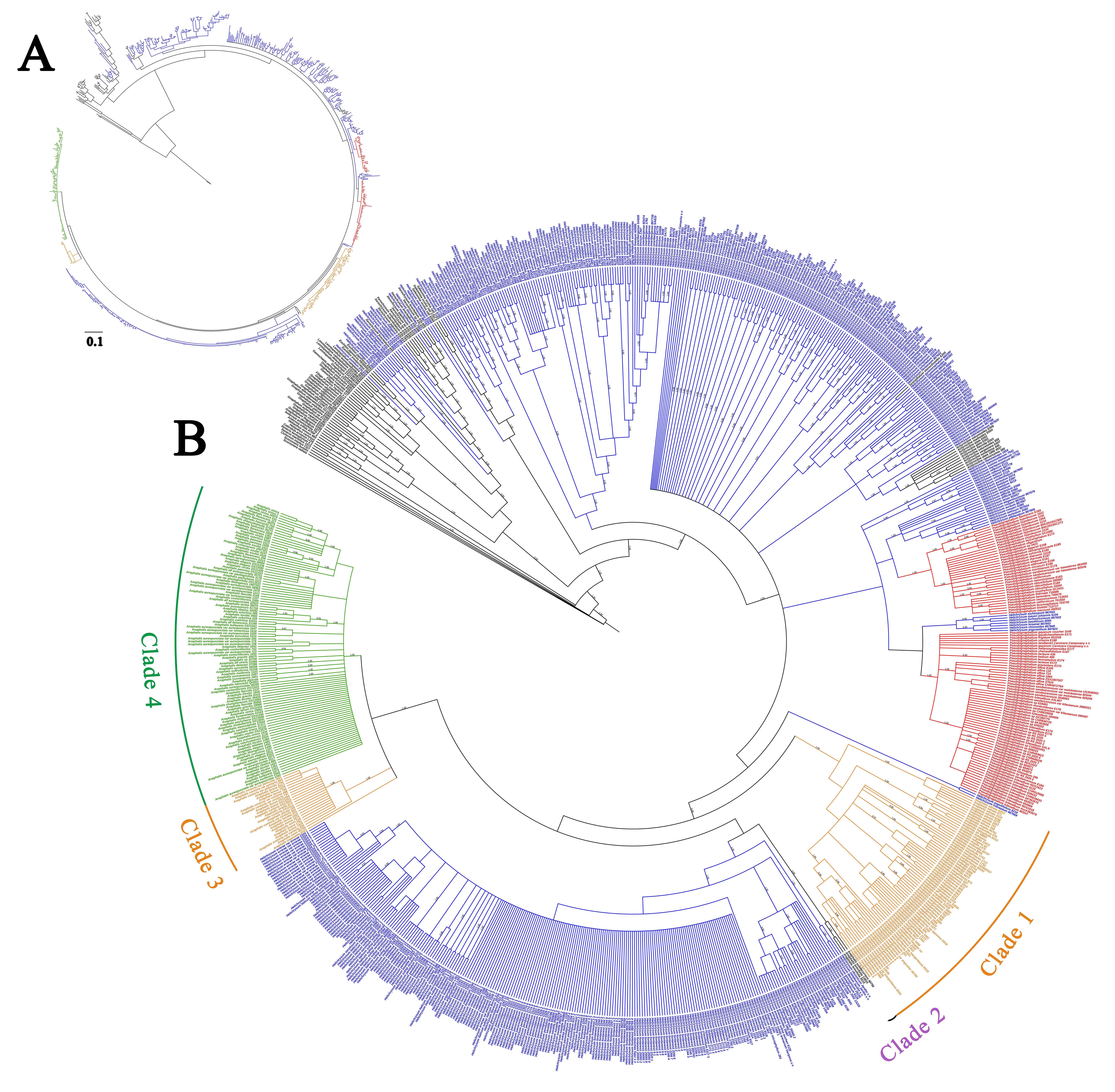

Supplement: Supplementary Figure 5 — Phylogenetic tree of the HAP clade and its closely related genera is inferred from ETS sequences using BI method. (A) Topologies. (B) The phylogenetic tree shows posterior probabilities of BI at each node. Posterior probabilities higher than 0.90 are indicated on branches. [file Image_5.jpeg]

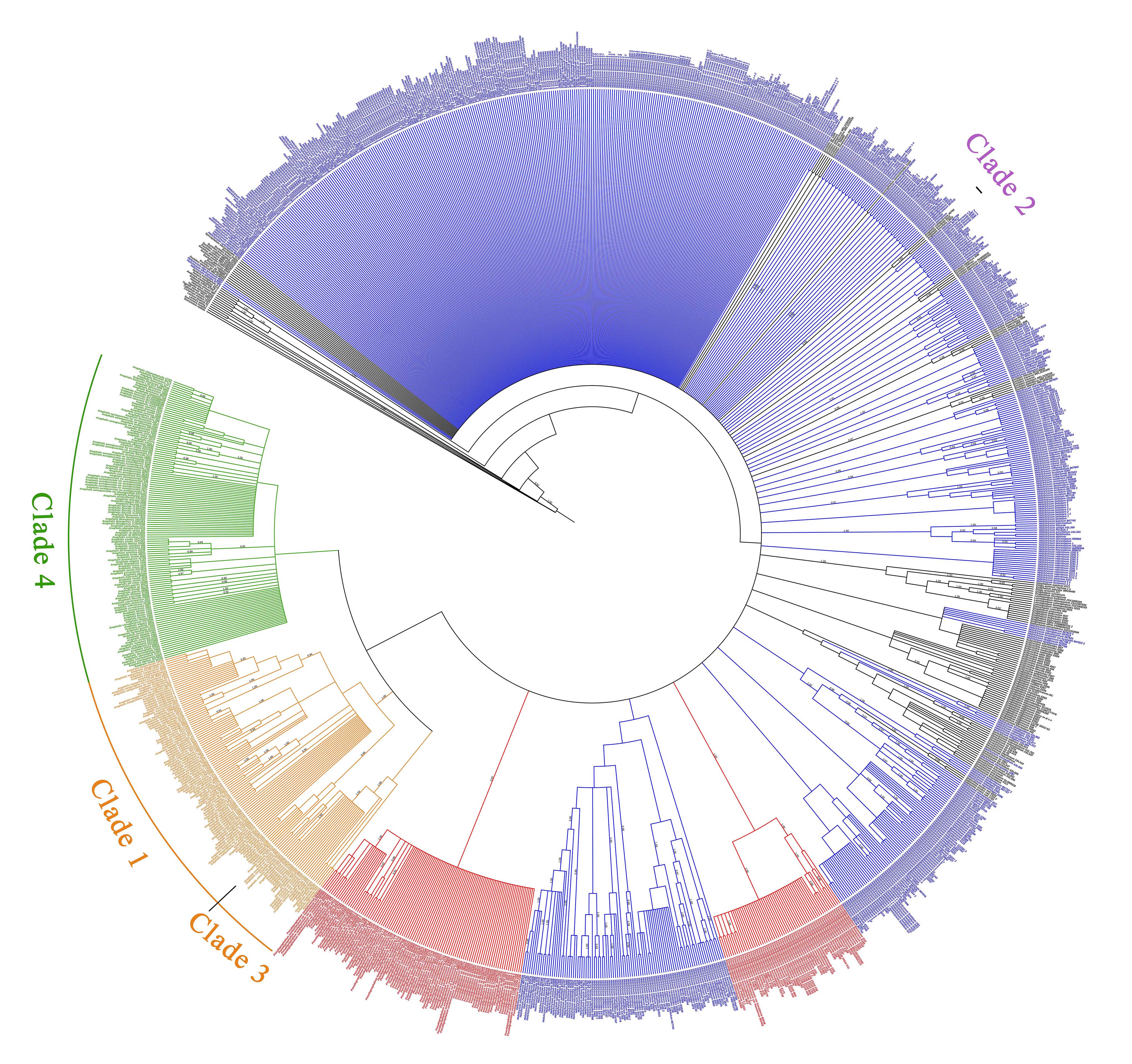

Supplement: Supplementary Figure 6 — Phylogenetic tree of the HAP clade and its closely related genera is inferred from the concatenated sequences of ITS and ETS using BI method. The posterior probabilities of BI are showed at each node. Posterior probabilities higher than 0.90 are indicated on branches. [file Image_6.jpeg]
