# Supplementary material for: Phylogeny, biogeography, and character evolution of Anaphalis (Gnaphalieae, Asteraceae)
Source: Front Plant Sci. 2024 Feb 7;15:1336229. doi: 10.3389/fpls.2024.1336229 (PMC10879626; doi:10.3389/fpls.2024.1336229)
Supplement: Supplementary file 11 [file Table_1.docx]

**Supplementary Table S1** **|** Accession numbers of cp genomes from NCBI database

| Taxa | Accession numbers |
| --- | --- |
| *Ambrosia artemisiifolia* | MF362689 |
| *Ambrosia trifida* | MG029118 |
| *Anaphalis margaritacea* var*. yedoensis* | LC656264 |
| *Anaphalis sinica* | KX148081 |
| *Calendula arvensis* | ON641308 |
| *Cichorium intybus* | MK569377 |
| *Filago arvensis* | OP963955 |
| *Filago arvensis* | OP963956 |
| *Gamochaeta coarctata* | MK570596 |
| *Gamochaeta pensylvanica* | OP963958 |
| *Helianthus annuus* | MK341450 |
| *Helianthus carnosus* | OK216126 |
| *Helichrysum italicum* | MK089797 |
| *Helichrysum italicum* subsp*. picardii* | ON641360 |
| *Helichrysum italicum* subsp*. picardii* | ON641306 |
| *Helichrysum italicum* subsp*. picardii* | ON641295 |
| *Jacobaea maritima* | OL960706 |
| *Lactuca praevia* | ON782561 |
| *Lactuca sativa* | AP007232 |
| *Leontopodium calocephalum* | OP963967 |
| *Leontopodium campestre* | OP963969 |
| *Leontopodium delavayanum* | OP963972 |
| *Leontopodium franchetii* | OP963975 |
| *Leontopodium leontopodioides* | OP963984 |
| *Pseudognaphalium affine* | OL894240 |
| *Pseudognaphalium affine* | MK570595 |
| *Pseudognaphalium affine* | MN541094 |
| *Pseudognaphalium californicum* | MK570608 |
| *Pseudognaphalium luteoalbum* | MT271604 |
| *Pseudognaphalium luteoalbum* | MK570605 |
| *Pseudognaphalium luteoalbum* | MK570609 |
| *Pseudognaphalium oligandrum* | MK570591 |
| *Pseudognaphalium sandwicensium* | MK570594 |
| *Pseudognaphalium sandwicensium* | MK570610 |
| *Pseudognaphalium sandwicensium* var*. hawaiiense* | MK570593 |
| *Pseudognaphalium sandwicensium* var*. molokaiense* | MK570592 |
| *Pseudognaphalium sandwicensium* var*. molokaiense* | MK771096 |
| *Pseudognaphalium sp.* | MK570598 |
| *Pseudognaphalium sp.* | MK421603 |
| *Pseudognaphalium sp.* | MK570604 |
| *Pseudognaphalium sp.* | MK570600 |
| *Pseudognaphalium sp.* | MK570601 |
| *Pseudognaphalium sp.* | MK570603 |
| *Pseudognaphalium sp.* | MK570606 |
| *Pseudognaphalium sp.* | MK570599 |
| *Pseudognaphalium sp.* | MK570607 |
| *Pseudognaphalium sp.* | MK570597 |
| *Pseudognaphalium sp.* | MK570602 |
| *Senecio keniophytum* | MH483946 |
| *Tagetes lemmonii* | OL467320 |
| *Tagetes minuta* | MZ198507 |
