# Supplementary material for: Phylogeny, biogeography, and character evolution of Anaphalis (Gnaphalieae, Asteraceae)
Source: Front Plant Sci. 2024 Feb 7;15:1336229. doi: 10.3389/fpls.2024.1336229 (PMC10879626; doi:10.3389/fpls.2024.1336229)
Supplement: Supplementary file 13 [file Table_3.docx]

**Supplementary Table S3 |** The codes of distribution areas and characters used in biogeographic and morphological analysis.

| Taxa | Regions^1^ | Achene micro-morphology^2^ | Leaf base^3^ |
| --- | --- | --- | --- |
| *Anaphalis acutifolia 31928* | D | R | C |
| *A. adnata 4748* | DEF | A | C |
| *A. aureopunctata 38255* | DF | L | B |
| *A. aureopunctata 38963* | DF | L | B |
| *A. aureopunctata 39694* | DF | L | B |
| *A. aureopunctata* var. *plantaginifolia DS13531* | DF | L | B |
| *A. aurora 8065* | BDE | A | C |
| *A. bicolor 36420* | D | L | B |
| *A. brevifolia 13833* | E | A | B |
| *A. brevifolia 5515* | E | A | B |
| *A. bulleyana DS11451* | DF | L | B |
| *A. busua DS11458* | BDEF | R | B |
| *A. busua DS13351* | BDEF | R | B |
| *A. cavei 228* | D | A | A |
| *A. cavei 46759* | D | A | A |
| *A. chlamydophylla 37391* | D | L | B |
| *A. cinerascens 1907351* | D | L | B |
| *A. contorta DS11511* | BDEF | R | D |
| *A. contorta DS13552* | BDEF | R | D |
| *A. contortiformis DS11523* | D | L | B |
| *A. delavayi 37286* | D | L | B |
| *A. elegans DS11438* | D | L | B |
| *A. elegans DS13580* | D | L | B |
| *A. flaccida 35858* | D | L | B |
| *A. flavescens 39275* | DF | L | B |
| *A. gracilis 29966* | D | L | B |
| *A. hancockii 39171* | DF | L | B |
| *A. hancockii QH001* | DF | L | B |
| *A. hellwigii 5889* | G | A | C |
| *A. hymenolepis 41543* | D | L | B |
| *A. lactea 39074* | D | L | B |
| *A. larium DS11434* | D | L | B |
| *A. likiangensis 1907201* | D | L | B |
| *A. margaritacea 150802037* | ADEF | R | C |
| *A. margaritacea 40183* | ADEF | R | C |
| *A. margaritacea* var. *angustior 46740* | ADEF | R | C |
| *A. margaritacea* var. *yedoensis LC656264* | F | R | C |
| *A. muliensis DS11436* | D | A | B |
| *A. nepalensis 41939* | BDF | R | C |
| *A. nubigena 62* | BDF | R | C |
| *A. pachylaena DS13590* | D | L | B |
| *A. pannosa DS11433* | D | L | B |
| *A. racemifera 470* | BD | A | C |
| *A. rhododactyla DS13591* | D | L | B |
| *A. roseoalba 46757* | BD | A | C |
| *A. royleana 5617* | DF | L | C |
| *A. sinica 29691* | DF | L | B |
| *A. sinica KX148081* | DF | L | B |
| *A. souliei DS13576* | D | L | B |
| *A. stenocephala 37044* | D | A | B |
| *A. subdecurrens 46756* | E | A | C |
| *A. suffruticosa 33158* | D | A | B |
| *A. surculosa 36266* | D | L | B |
| *A. szechuanensis 37371* | D | A | B |
| *A. tenella 67A* | BDEF | A | C |
| *A. tibetica 32092* | D | A | B |
| *A. triplinervis 35925* | BD | R | C |
| *A. virens 32134* | D | L | B |
| *A. virgata 1375* | BD | A | C |
| *A. virgata 46761* | BD | A | C |
| *A. viridis DS11428* | D | A | B |
| *A. xylorhiza 41631* | D | L | B |
| *A. yunnanensis 41728* | D | L | B |
| *Helichrysum argyrophyllum L001* | C | A | A |
| *H. argyrophyllum L002* | C | A | A |
| *H. italicum L003* | BC | A | A |
| *H. italicum MK089797* | BC | A | A |
| *H. italicum* subsp. *picardii ON641295* | BC | A | A |
| *H. italicum* subsp. *picardii ON641306* | BC | A | A |
| *H. italicum* subsp. *picardii ON641360* | BC | A | A |
| *Pseudognaphalium affine 37610* | BDEF | A | A |
| *P. affine MK570595* | BDEF | A | A |
| *P. affine MN541094* | BDEF | A | A |
| *P. affine OL894240* | BDEF | A | A |
| *P. affine ZSX1907007* | BDEF | A | A |
| *P. californicum MK570608* | A | A | A |
| *P. hypoleucum 2234* | BDEF | A | A |
| *P. hypoleucum BNU2019SC273* | BDEF | A | A |
| *P. hypoleucum BNU2019XZ300* | BDEF | A | A |
| *P. luteoalbum 299* | BCDEFG | A | A |
| *P. luteoalbum MK570605* | BCDEFG | A | A |
| *P. luteoalbum MK570609* | BCDEFG | A | A |
| *P. luteoalbum MT271604* | BCDEFG | A | A |
| *P. oligandrum MK570591* | C | A | A |
| *P. sandwicensium MK570594* | A | A | A |
| *P. sandwicensium MK570610* | A | A | A |
| *P. sandwicensium* var. *hawaiiense MK570593* | A | A | A |
| *P. sandwicensium* var. *molokaiense MK570592* | A | A | A |
| *P. sandwicensium* var. *molokaiense MK771096* | A | A | A |
| *P. sp. MK421603* | BCDEFG | A | A |
| *P. sp. MK570598* | BCDEFG | A | A |
| *P. sp. MK570603* | BCDEFG | A | A |
| *P. sp. MK570606* | BCDEFG | A | A |
| *P. sp. MK570597* | BCDEFG | A | A |
| *P. sp. MK570599* | G | A | A |
| *P. sp. MK570600* | BCDEFG | A | A |
| *P. sp. MK570601* | BCDEFG | A | A |
| *P. sp. MK570602* | BCDEFG | A | A |
| *P. sp. MK570604* | BCDEFG | A | A |
| *P. sp. MK570607* | BCDEFG | A | A |

Note:

1 Distribution areas: A, N America; B, Europe, W Asia; C, Africa; D, SW China; E, S Asia; F, E and SE Asia; G, New Guinea, Australia, New Zealand, Pacific islands.

2 Achene micro-morphology: A, No data; L, Ligulate protuberant; R, Reticulate-claviform.

3 Leaf base: A, No data; B, Decurrent; C, Non-decurrent; D, Cordate.
