# Supplementary material for: Phylogeny, biogeography, and character evolution of Anaphalis (Gnaphalieae, Asteraceae)
Source: Front Plant Sci. 2024 Feb 7;15:1336229. doi: 10.3389/fpls.2024.1336229 (PMC10879626; doi:10.3389/fpls.2024.1336229)
Supplement: Supplementary file 14 [file Table_4.docx]

**Supplementary Table S4 |** Sequences characteristics and models selected in ML and BI phylogenetic analyses of different data set

|  | Aligned length (bp) | Variable sites | |  | Parsimony informative sites | | Model in ML | Model in BI |
| --- | --- | --- | --- | --- | --- | --- | --- | --- |
|  |  | Numbers | % |  | Numbers | % |  |  |
| Complete cp genome | 182,886 | 23,392 | 12.79 |  | 15,937 | 8.71 | TVM+F+R10 | GTR+F+I+G4 |
| ITS | 719 | 510 | 70.93 |  | 422 | 58.69 | GTR+F+I+G4 | GTR+F+I+G4 |
| ETS | 680 | 518 | 76.18 |  | 444 | 65.29 | GTR+F+R3 | GTR+F+I+G4 |
| Concatenated sequences of ITS and ETS | 1399 | 1028 | 73.48 |  | 866 | 61.90 | GTR+F+I+G4 | GTR+F+I+G4 |
